# Network analysis combined with experimental assessment to explore the therapeutic mechanisms of New Shenqi Pills formula targeting mitochondria on senile diabetes mellitus

**DOI:** 10.3389/fphar.2024.1339758

**Published:** 2024-06-12

**Authors:** YueYing Zhang, Yang Zhou, ZhiGe Wen, HaoShuo Wang, Shan Zhang, Qing Ni

**Affiliations:** ^1^ Guang’anmen Hospital, China Academy of Chinses Medicine Sciences, Beijing, China; ^2^ Beijing University of Chinese Medicine, Beijing, China

**Keywords:** senile diabetes, New Shenqi Pills, network analysis, vivo experiments, oxidative stress, mitochondrial cristae structure

## Abstract

**Background:**

The escalation of global population aging has accentuated the prominence of senile diabetes mellitus (SDM) as a consequential public health concern. Oxidative stress and chronic inflammatory cascades prevalent in individuals with senile diabetes significantly amplify disease progression and complication rates. Traditional Chinese Medicine (TCM) emerges as a pivotal player in enhancing blood sugar homeostasis and retarding complication onset in the clinical management of senile diabetes. Nonetheless, an evident research gap persists regarding the integration of TCM’s renal tonification pharmacological mechanisms with experimental validation within the realm of senile diabetes therapeutics.

**Aims:**

The objective of this study was to investigate the mechanisms of action of New Shenqi Pills (SQP) in the treatment of SDM and make an experimental assessment.

**Methods:**

Network analysis is used to evaluate target pathways related to SQP and SDM. Mitochondrial-related genes were obtained from the MitoCarta3.0 database and intersected with the common target genes of the disease and drugs, then constructing a protein-protein interaction (PPI) network making use of the GeneMANIA database. Representative compounds in the SQP were quantitatively measured using high performance liquid chromatography-tandem mass spectrometry (HPLC-MS/MS) to ensure quality control and quantitative analysis of the compounds. A type 2 diabetes mice (C57BL/6) model was used to investigate the pharmacodynamics of SQP. The glucose lowering efficacy of SQP was assessed through various metrics including body weight and fasting blood glucose (FBG). To elucidate the modulatory effects of SQP on pancreatic beta cell function, we measured oral glucose tolerance test (OGTT), insulin histochemical staining and tunel apoptosis detection, then assessed the insulin-mediated phosphoinositide 3-kinase (PI3K)/protein kinase A (Akt)/glycogen synthase kinase-3β (GSK-3β) pathway in diabetic mice via Western blotting. Additionally, we observe the structural changes of the nucleus, cytoplasmic granules and mitochondria of pancreatic islet β cells.

**Results:**

In this investigation, we identified a total of 1876 genes associated with senile diabetes, 278 targets of SQP, and 166 overlapping target genes, primarily enriched in pathways pertinent to oxidative stress response, peptide response, and oxygen level modulation. Moreover, an intersection analysis involving 1,136 human mitochondrial genes and comorbidity targets yielded 15 mitochondria-related therapeutic targets. Quality control assessments and quantitative analyses of SQP revealed the predominant presence of five compounds with elevated concentrations: Catalpol, Cinnamon Aldehyde, Rehmanthin D, Trigonelline, and Paeonol Phenol. Vivo experiments demonstrated notable findings. Relative to the control group, mice in the model group exhibited significant increases in body weight and fasting blood glucose levels, alongside decreased insulin secretion and heightened islet cell apoptosis. Moreover, β-cells nuclear condensation and mitochondrial cristae disappearance were observed, accompanied by reduced expression levels of p-GSK-3β protein in islet cells (*p* < 0.05 or *p* < 0.01). Conversely, treatment groups administered SQP and Rg displayed augmented expressions of the aforementioned protein markers (*p* < 0.05 or *p* < 0.01), alongside preserved mitochondrial cristae structure in islet β cells.

**Conclusion:**

Our findings suggest that SQP can ameliorate diabetes by reducing islet cell apoptosis and resist oxidative stress. These insulin-mediated PI3K/AKT/GSK-3β pathway plays an important regulatory role in this process.

## 1 Introduction

Presently, there is a notable global demographic shift toward an aging population, with projections indicating that the proportion of elderly individuals worldwide will rise from the current 15%–25% by 2050. This demographic trend underscores the increasing significance of addressing senile diabetes mellitus (SDM) as a focal point in diabetes prevention and management strategies. Among the elderly, diabetes often manifests with insulin secretion deficits and a heightened susceptibility to hypoglycemia, alongside a spectrum of comorbidities affecting the cardiovascular, cerebrovascular, and renal systems, compounded by conditions such as hypertension and hyperlipidemia. Recent literature underscores the complexity of managing diabetes in older adults, highlighting the challenge of achieving therapeutic efficacy with monotherapeutic approaches. Consequently, there is a growing imperative for the implementation of comprehensive, multidisciplinary management strategies to mitigate disability and mortality rates in elderly diabetic populations. Consequently, there is burgeoning interest in exploring Traditional Chinese Medicine (TCM) formulations with multitarget therapeutic profiles and integrating TCM with conventional western medical modalities as promising avenues for addressing elderly diabetes.

One of the causes of SDM is considered to be spleen and kidney deficiency in TCM theory. The treatment strategy is often based on syndrome differentiation and treatment, focusing on strengthening the spleen and kidneys. Notable among the TCM preparations are Shenqi Pills, known for their kidney-tonifying and qi-tonifying effects. The formula of New Shenqi Pills (SQP), based on the traditional “Jingui Shenqi Pills”, has evolved to include Codonopsis (Campanulaceae; Codonopsis pilosula), Astragalus (Faboideae; Astragalus membranaceus), Purslane (Portulacaceae; Portulaca oleracea), and Fenugreek (Fabaceae Papilioideae; Trigonella foenum-graecum L.). Clinical studies have shown that Shenqi Pills have good hypoglycemic effects in the treatment of diabetes. Pharmacological studies and related animal experimental studies have shown that Shenqi Pills can regulate glucose homeostasis and improve insulin resistance by intervening in inflammation-related pathways. The relevant mechanism may be related to affecting the expression of PI3K/AKT pathway. However, the mechanism of action of Shenqi Pills on SDM has not been clearly verified. Based on this, we modified Shenqi pills and investigated their mechanism of action in the treatment of SDM. Given the multifaceted nature of TCM components and targets, elucidating their potential therapeutic mechanisms remains a formidable challenge that needs to be explored through network analysis and experimental exploration.

**FIGURE 1:**
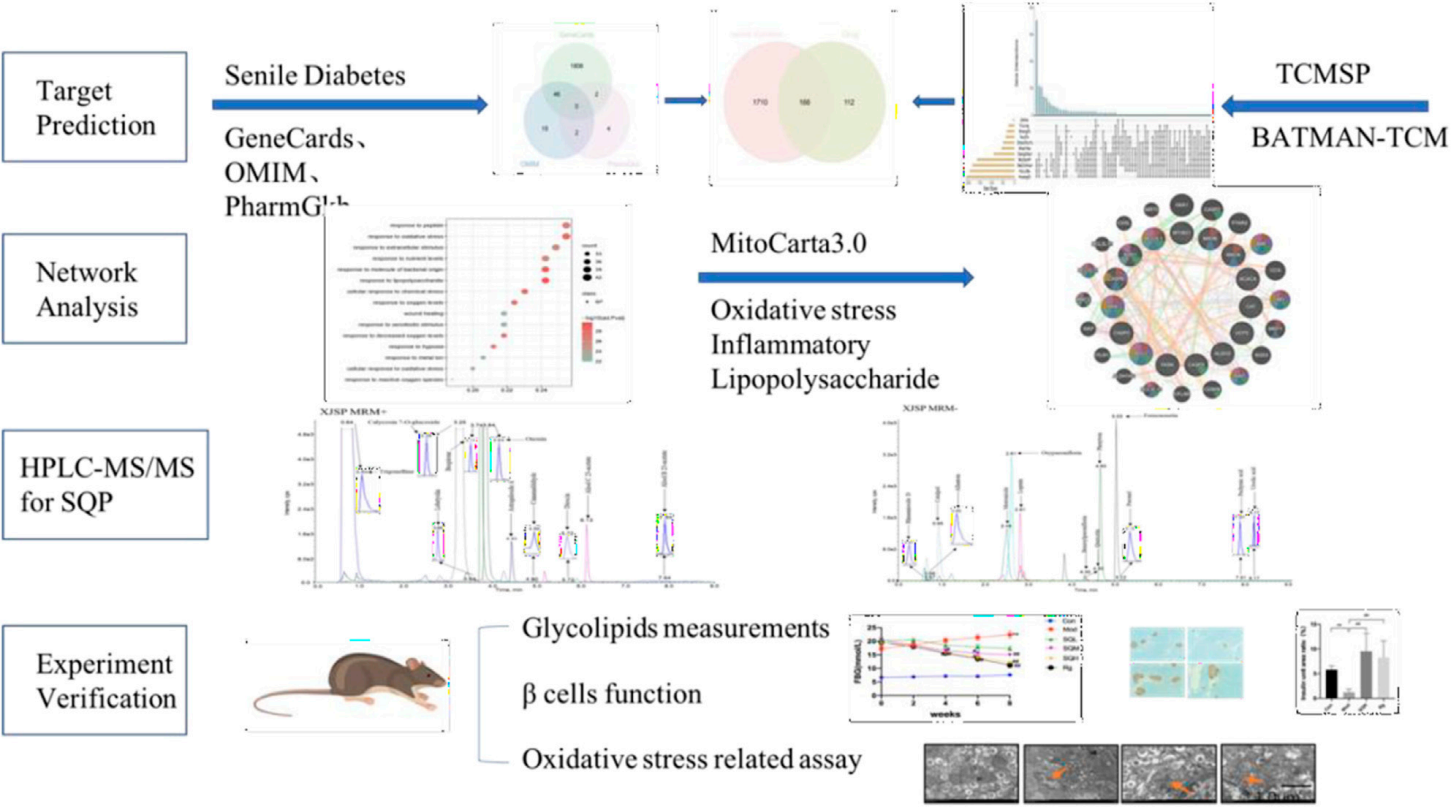
Flowchart of ideas for this study..

Network analysis offers a systematic framework for elucidating drug-disease interactions through the delineation of compound-protein/gene-disease networks. By probing these networks, one can discern the modulatory effects of SQP on SDM, while concurrently forecasting the impacts of pharmacological constituents on pivotal targets and associated pathways. Moreover, the employment of high-performance liquid chromatography coupled with mass spectrometry (HPLC-MS/MS) facilitates the precise quantification of molecular entities. This analytical approach is further reinforced by *in vivo* investigations employing murine models, which serve to validate the therapeutic efficacy of SQP via serological assays, Western blotting, and electron microscopy analyses (Figure 1). This integrative methodology furnishes substantive empirical insights into the therapeutic potential of SQP in SDM management, fostering the advancement of multi-target therapeutic strategies.

## 2 Materials and methods

### 2.1 Chemicals and reagents

The SQP granules were purchased from the Pharmacy Department of Guang’anmen Hospital (Beijing, China, May to August 2023). Drug composition: *Rehmannia glutinosa* [Scrophulariaceae Rehmannia genus; *Radix Rehmanniae root tuber*], *Rhizoma dioscoreae* [Dioscoreaceae; *Dioscorea dried rhizome*], *Cornus officinalis* [Dogwood family; *Dogwood dry ripe pulp*], *Poria* [Polyporaceae; *Poria cocos dried sclerotia*], *Alisma orientalis* [Alismataceae; *Alisma dried tubers*], *Peony bark* [Ranunculaceae; *Peony dry root bark*], *Cinnamomi Ramulus* [Lauraceae; *Dried twigs of cinnamon*], *Codonopsis pilosula* [Platycodonaceae; *Dried root of Codonopsis pilosula*], *Astragalus membranaceus* [Leguminosae; *Dried roots of Astragalus membranaceus*], *Trigonella foenum-graecum* [Leguminosae; *Fenugreek dry mature seeds*], *Purslane* [Portulacaceae; *Dry aerial parts of purslane*] (Table 1). Rosiglitazone (Chengdu Hengrui Pharmaceutical Co. Ltd., China. H20030569). High-fat diet including 58 kcal% fat (mostly derived from saturated fatty acids), 25 kcal% protein, and 17 kcal% carbohydrates (D12492) was obtained from Research Diets (New Brunswick, NJ, United States of America). STZ (S0130; CAS Number: 18,883–66–4) was obtained from Sigma (Livonia, MI, United States of America); LY294002 (ab120243; CAS number: 154,447–36–6), a PI3K inhibitor that can suppress phosphorylated (p)Akt expression, GSK-3β(ab93926) were obtained from Abcam (Cambridge, United Kingdom). GAPDH (ab8245), PI3K (4257), pPI3K (4228S), AKT (4691), pAKT (4060S), and pGSK -3β(ser-9) (5558T) were obtained from CST (Boston, MA, United States of America); High glucose/palmitic acid (Glu/PA; KT002) was obtained from Kunchuang (Xi’an, China); an annexin V-FITC/Pro-pidium iodide (PI) detection kit (KGA108) was obtained from KeyGEN BioTECH (Beijing, China).

**TABLE 1:** SQP Dosage (dosage per dose of medication).

Botanical drug	Unit (g)	Botanical drug	Unit (g)
Rehmannia glutinosa	24	Peony bark	9
Rhizoma dioscoreae	12	Cinnamomi ramulus	3
Cornus officinalis	12	Codonopsis pilosula	9
Poria	9	Astragalus membranaceus	12
Alisma orientalis	9	Trigonella foenum-graecum	6
Purslane	9		

### 2.2 Access to SQP and SDM targets

Use “senile diabetes” as the keyword to search the GeneCards database (https://www.genecards.org/), set Score≥1, OMIM database (https://omim.org/), and PharmGkb database (https://www.pharmgkb.org/), remove duplicates and then take the union to obtain the disease genes of SDM.

Search the Traditional Chinese Medicine Systems Pharmacology Database and Analysis Platform (TCMSP, https://old.tcmsp-e.com/index.php) to collect SQP Chinese yam, dogwood, poria, Alisma, paeonol, cassia twig, astragalus, dangshen, For medicinal ingredients such as fenugreek and purslane, search the BATMAN-TCM database (http://bionet.ncpsb.org.cn/batman-tcm/) to collect the main chemical ingredients of raw materials that are not included in the TCMSP database and take them orally Bioavailability (OB) ≥ 30% and drug-likeness (DL) ≥ 0.18 are the screening conditions. Combined with literature research, ingredients that do not meet the screening conditions but have clear pharmacological effects are included as candidates. Use the TCMSP database to search for verified component targets in the active ingredients obtained, import the obtained targets into the Uniprot database (https://www.uniprot.org/), limit the species to human, and correct the target gene name to its official name. The common target genes of SDM and SQP were obtained through R software.

### 2.3 Analysis of intersectional target pathways and functional enrichment

Use the R software “clusterProfiler” package, set the species to human origin (*Homo sapiens*), and conduct Biological Process (BP) Gene Ontology (GO) enrichment analysis on the common target genes of SDM and SQP. The corrected *p*-value < 0.05 was used as the filtering condition.

### 2.4 Access to mitochondria-related gene

Mitochondria-related genes were obtained through the MitoCarta3.0 database (https://www.broadinstitute.org/mitocarta/mitocarta30-inventory-mammalian-mitochondrial-proteins-and-pathways). The database performs mass spectrometry analysis of mitochondria isolated from 14 tissues, evaluates protein localization by large-scale GFP tagging/microscopy, and integrates the results using a Bayesian algorithm with six other genomic datasets of mitochondrial localization. Contains 1,136 human mitochondrial genes and 1,140 mice mitochondrial genes^7.^

### 2.5 Construction of mitochondria related genes-disease target protein interaction network

The mitochondria-related genes were intersected with the comorbid disease targets of SDM and SQP to obtain mitochondria-related drug treatment targets. The GeneMANIA database (http://genemania.org/) was used to select the top five functions.

### 2.6 Animal experiment

#### 2.6.1 Quality control of SQP using high-performance liquid chromatography/mass spectrometry (HPLC-MS/MS)

HPLC-MS/MS (Agilent, Santa Clara, CA, United States of America) was used to quantitatively determine the levels of representative compounds in SQP. Mass spectrometry measurements were performed using a Sciex API 4000 Qtrap MS system equipped with a Turbo Ionspray interface (Applied Biosystems, Foster City, CA, United States of America). The samples were analyzed in either positive or negative electrospray ionization mode and monitored in the multiple reactions monitoring mode. High-purity nitrogen was used as the curtain gas, ion source gas 1, and ion source gas 2, with ow rates of 30, 60, and 60 psi, respectively. The spray voltage was ±4.5 kV, and the capillary temperature was set to 600°C.

#### 2.6.2 Experimental models and pharmacotherapy

Male C57BL/6 mice (*n* = 70)aged 6–8 weeks were purchased from Beijing Vital River Laboratory Animal Technology Company (certificate number SCXK 2019–0010, Beijing, China). They were randomly divided into a control (con) group (*n* = 10)and a high-fat diet (HFD) group (*n* = 60). All animals were housed in groups of four to six animals and maintained under a 12 h light–dark cycle at 20◦C–24◦C with free access to food and water. Mice in the HFD group were given a one-time intraperitoneal injection of 30 mg/kg STZ and blood was collected from the tail tip 72 h later to measure fasting blood glucose (FBG). We selected mice with FBG ≥16.7 mmol/L as successful SDM modeling mice, and divided them into six groups according to body weight using balanced random sampling: model, (Mod), shenqi low (SQL), shenqi middle (SQM), shenqi high (SQH), rosiglitazone (Rg) and SQH + PI3K inhibitor LY294002 (S + LY; 29.6 g/kg SQP+25 mg/kg LY294002 intraperitoneally, 20 min before SQP gavage) groups.

**FIGURE 2:**
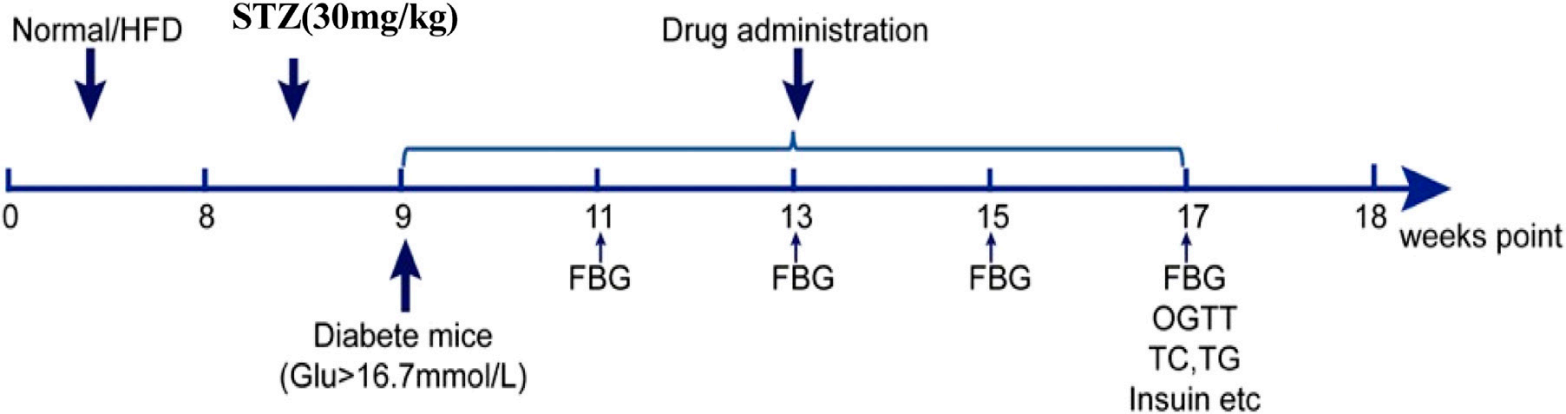
SDM mice modeling and medication timeline.

According to a mouse-to-human drug conversion ratio of 9:1, the dose (g/kg) of SQP for each mice was calculated as follows: 9 × the daily adult dose (114 g of crude drug)/the average adult weight (70 kg) = 14.8 g (crude drug)/kg. We designated this as the medium-dose group according to a previous study. The low-dose group was set at 7.4 g/kg, and the high-dose group was set at 29.6 g/kg. The modern drug group was given rosiglitazone 0.52 mg/kg/d by gavage, and the normal group was given an equal volume of normal saline by gavage, once a day for eight consecutive weeks (Figure 2).

#### 2.6.3 Serum parameter measurements

FBG was measured at 0, 2, 4, 6, and 8 weeks after administration. At the end of 8 weeks, the levels of glycated albumin (GA), cholesterol (CHO), triglyceride (TG), high density lipoprotein (HDL), low density lipoprotein (LDL), and free fatty acids (FFA) were measured.

#### 2.6.4 β cells function

Oral Glucose Tolerance Test Mice in each group were fasted for 10 h at the end of 8 weeks after administration, and blood glucose values were measured again at 30, 60, and 120 min after glucose loading. Oral Glucose Tolerance Test (OGTT) curves were drawn based on the blood glucose values at each time point.

Insulin secretion assay The Fasting insulin (FINS) value of mice in each group at the end of 8 weeks was measured using an iodine [^125^I] insulin radioimmunoassay kit.

Prepare paraffin-embedded sections, dewax and hydrate the sections, inactivate endogenous enzymes, and microwave antigen retrieval. DAB chromogenic solution and hematoxylin staining solution were added in sequence, and 1% hydrochloric acid alcohol differentiation solution was used. After dehydration, transparency, and sealing, the stained sections were placed under an automatic scanner for scanning and observation.

β cells tunel apoptosis detection The paraffin sections were dewaxed, and proteinase K working solution was added to cover the tissue. After breaking the membrane, buffer was added to cover the tissue. After incubation at 37°C for 2 h, the nuclei were counterstained with DAPI. After sealing, the cells were observed under a fluorescence microscope and images were collected.

#### 2.6.5 β cells oxidative stress related assay

Transmission electron microscopy Observe the structural changes of the nucleus, cytoplasmic granules and mitochondria of pancreatic islet β cells. After the pancreatic tissue is peeled off, cut the pancreatic tail tissue into a size of 1 mm^3^ and fix it in 2.5% glutaraldehyde overnight. Change the PBS buffer the next day, fix it with osmic acid, perform gradient dehydration, infiltration and embedding, trimming, sectioning and staining, use transmission electron microscopy to observe mitochondrial structure and collect images.

Western blots Take the mice pancreatic tissue and quantitatively measure the protein concentration using the BCA method. After loading the sample for electrophoresis and electrotransfer, add the primary antibody and incubate at 4°C overnight. After washing the membrane, incubate the secondary antibody at room temperature for 120 min. After washing the membrane, use exposure solution to develop color. Imaging with a chemiluminescence imaging system, and using ImageJ software to count the gray value of the protein band and the corresponding gray value of the internal reference protein band. The gray value target band/gray value internal reference band is the relative content of the target protein contained in each sample. At least three samples were measured for each target gene.

### 2.7 Statistical analysis

SPSS 26.0 software was used for statistical analysis of the results, and GraphPad Prism eight software was used for graphing. The data obtained from the experiment are measurement data. Firstly, the normal distribution test is carried out. The independent sample *t*-test was used for comparison between two groups of data, and Levene’s method was used to test the homogeneity of variances in the data. If the variances were homogeneous, one-way analysis of variance was used to compare data between multiple groups. *p <* 0.05 means the difference is statistically significant, *p* < 0.01 means the difference is statistically significant.

## 3 Results

### 3.1 Results of database analysis

#### 3.1.1 Network analysis research shows that the target genes of SQP treatment of SDM related to oxidative stress response

**FIGURE 3:**
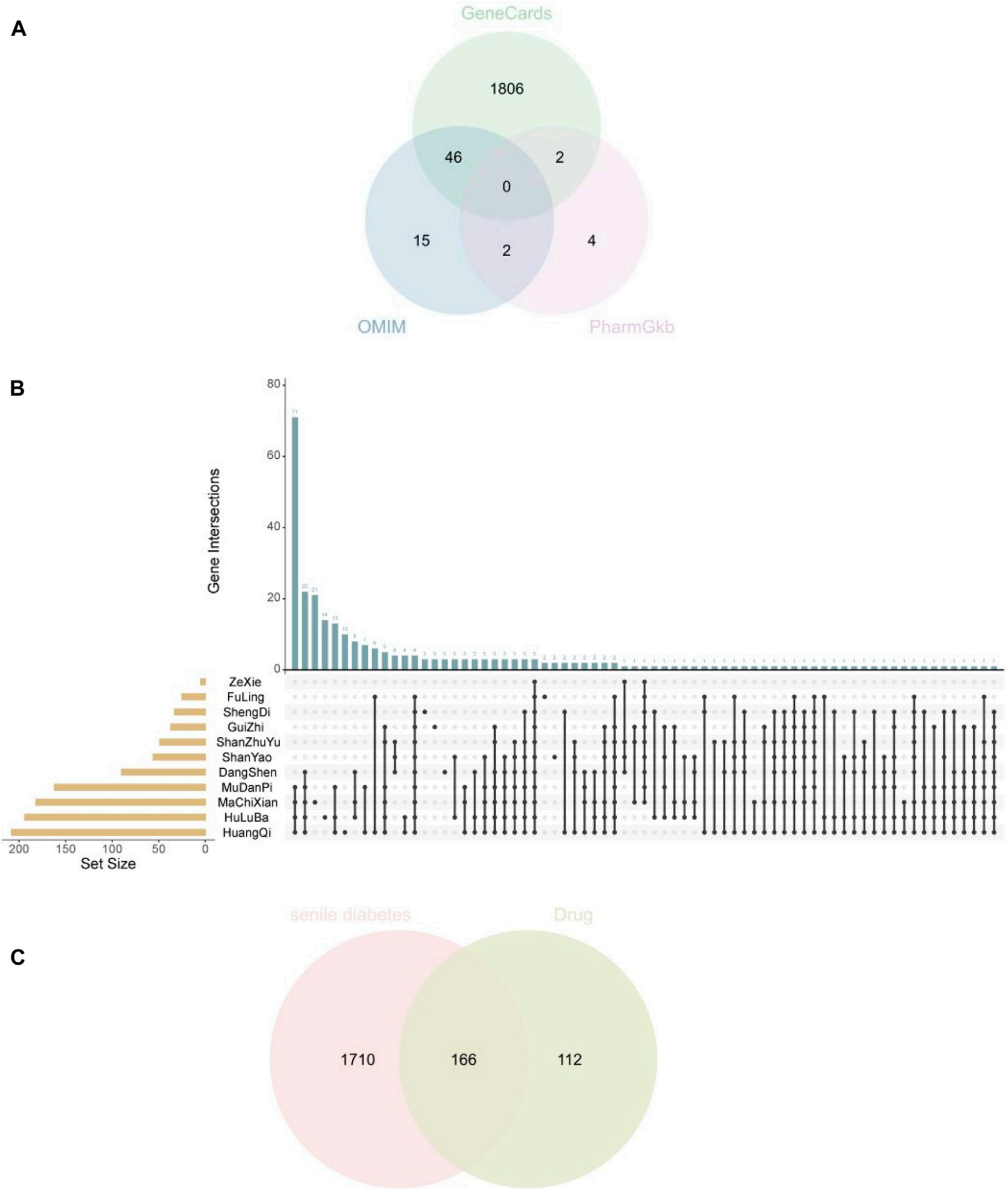
**(A)** Three databases obtain the disease genes of diabetes in the elderly **(B)** Drug target genes of Shenqi Pills **(C)** Common target genes of SDM and SQP.

SDM disease genes were obtained from the GeneCars database, OMIM database, and PharmGkb database, and 1854, 139, and 64 disease genes were obtained respectively. After merging the three, 1876 SDM disease genes were obtained by removing duplicates. Drug target genes were obtained through TCMSP and BATMAN-TCM databases, and a total of 278 SQP target genes were obtained. After obtaining the SDM and SQP target genes, they were intersected to obtain a total of 166 common target genes (Figures 3A–C).

**FIGURE 4:**
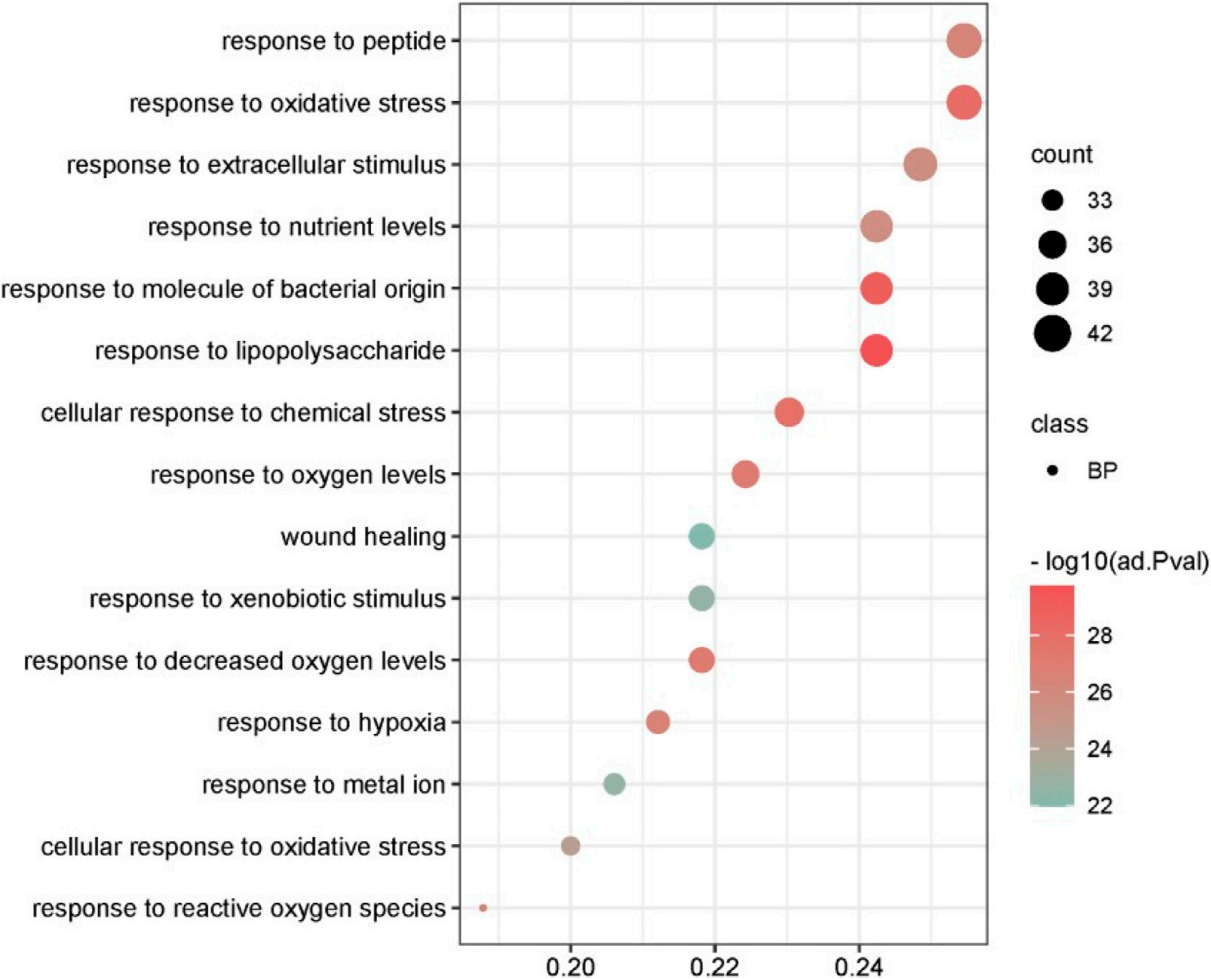
GO enrichment analysis of BP-related common target genes of SDM and SQP.

In order to elucidate the various biological functions and mechanisms of SQP in alleviating SDM, GO enrichment analysis was performed on 166 common target genes, using *p* < 0.05 as the selection condition. The BP results showed that the key cross-target genes of SQP in treating SQM were mainly enriched. It focuses on related biological processes such as oxidative stress response, inflammatory response, and response to lipopolysaccharide (Figure 4).

#### 3.1.2 Comorbid target genes are related to mitochondria combining with MitoCarta3.0 database

**FIGURE 5:**
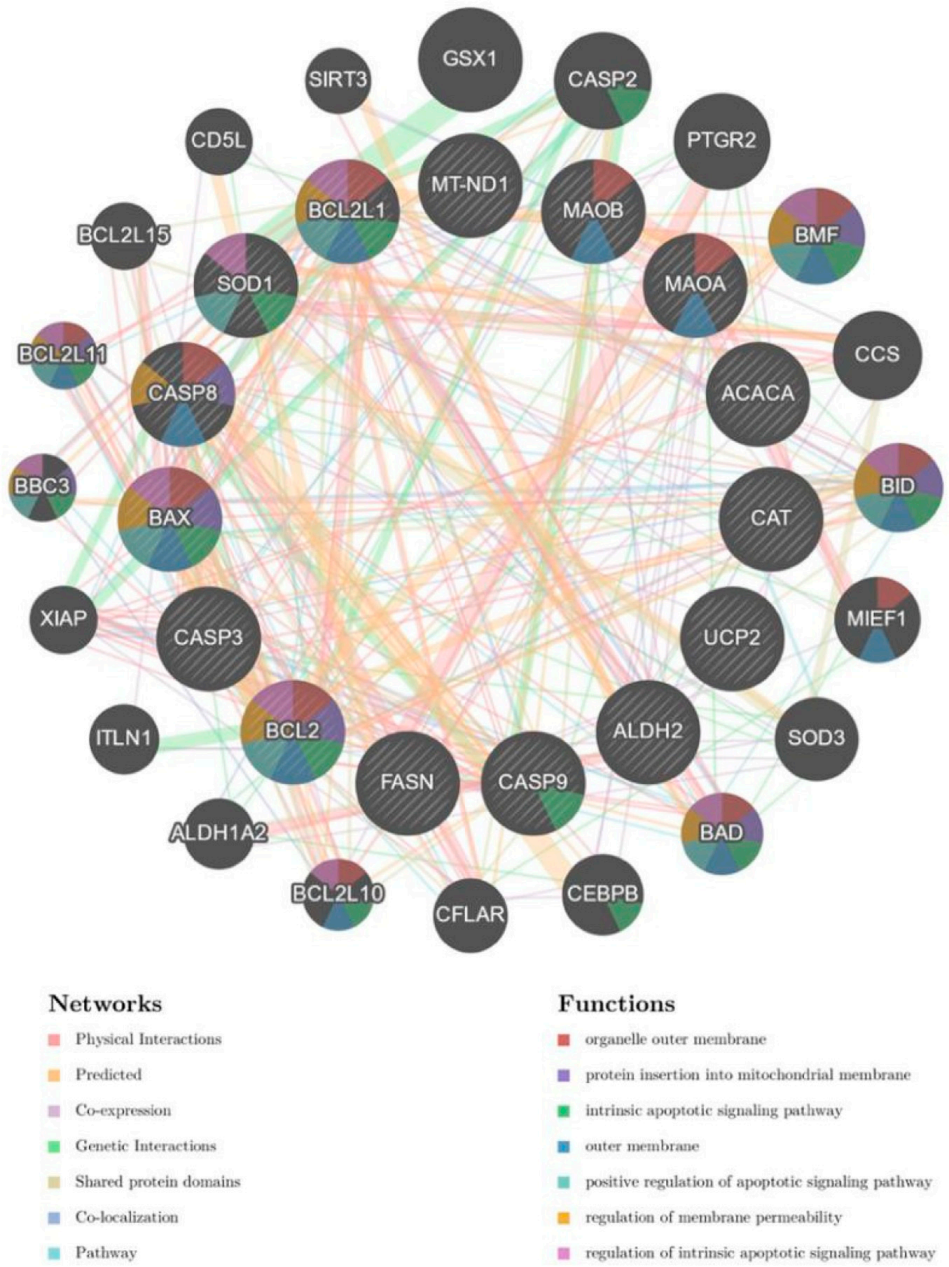
Mitochondria-related drug therapeutic target functions. (Nodes in the network diagram represent potential target genes, and connections between nodes refer to interactions between proteins. The node's size and color will be scaled using the “degree” value.

Combined with the results of GO enrichment analysis, we concluded that SQP may play a role in the treatment of SDM by improving the biological processes of oxidative stress and chronic inflammation. In order to furtherly verify the relevant biological molecular mechanisms, we obtained mitochondria-related genes through the MitoCarta3.0 database, and intersect with the comorbid targets of SDM and SQP, and use the GeneMANIA database to select the top five functions of the intersection genes, which are mitochondrial outer membrane, protein embedded in mitochondrial membrane, apoptosis signaling pathway, and positive regulation of apoptosis signaling pathways, regulation of mitochondrial membrane permeability, etc. The interacting targets were imported into the STRING database, and the network was further visualized and analyzed by Cytoscape 3.8.2 to construct a protein interaction map (Figure 5).

### 3.2 Results of animal experiment

#### 3.2.1 The main component of SQP has a protective effect on pancreatic β cells

**FIGURE 6:**
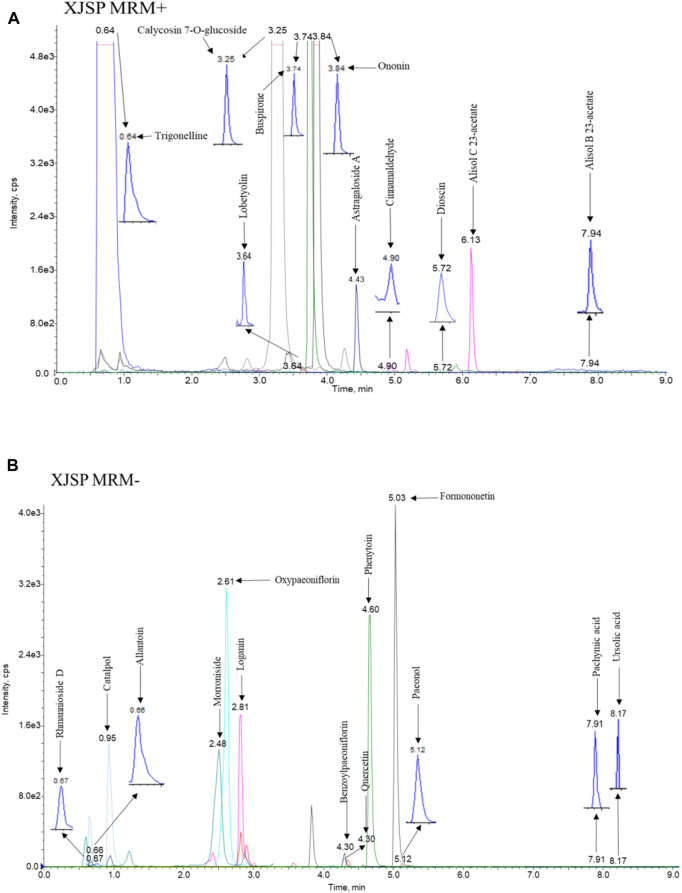
Representative chromatograms of 21 compounds in SQP. **(A)** Positive ion mode chromatogram **(B)** Negative ion mode chromatogram.

HPLC-MS/MS analysis was performed to determine the 21 compounds in SQP: rhmannioside D, catalpol, loganin, morroniside, ursolic acid, cinnamaldehyde, trigonelline, lobetyolin, alisol B 23-acetate, alisol C 23-acetate, allantoin, dioscin, calycosin 7-O-glucoside, formononetin, ononin, benzoylpaeoniflorin, oxypaeoniflorin, paeonol, astragaloside A, quercetin and pachymic acid content in the SQP granules. The chromatographic pro les of SQP are shown in Figures 6A, B. Mass spectrometry analysis of SQP showed that the top five compounds with higher concentrations in SQP were: catalpol, cinnamic aldehyde, digoxin D, trigonelline and paeonol, followed by iridoid glycosides (Table 2).

**TABLE 2:** Actual measured concentrations of 21 compounds in SQP.

Compounds	Concentrations (μg/g)			
	SQP 1–1	SQP 1–2	SQP 2–1	SQP 2–2
Rhmannioside D	490	458	361	449
Catalpol	5100	4730	4200	3990
loganin	308	292	323	264
Monogside	291	289	293	283
Ursolic acid	1.83	2.30	0.656	0.930
Cinnamaldehyde	615	741	864	964
Trigonelline	266	280	489	508
Lobetyolin	32.1	31.5	37.7	35.9
Alisol B 23-acetate	35.0	31.6	52.2	64.3
Alisol C 23-acetate	5.83	7.04	10.9	13.9
Allantoin	201	196	413	313
Dioscin	24.8	17.2	17.7	16.0
Calycosin 7-*O*-glucoside	52.3	55.4	61.0	64.5
Formononetin	10.6	9.95	18.9	18.4
Ononin	50.3	59.1	107	122
Benzoylpaeoniflorin	330	320	267	253
Oxypaeoniflorin	254	240	189	189
Paeonol	308	366	328	252
Astragaloside A	117	141	133	158
Quercetin	3.29	2.36	3.12	2.19
Poria acid	17.6	16.8	4.48	6.47

#### 3.2.2 SQP reduced body weight, and improved glucose homeostasis in diabetic mice

**FIGURE 7:**
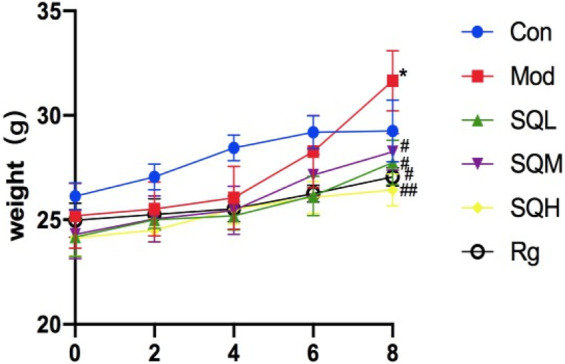
Body weight of mice in each group at different time points. (Compared with Con, *p < 0.05, **p < 0.01; Compared with Mod, ^#^ *p* < 0.05; ^##^ *P*<0.01).

Weight after the model is stable and before formal intervention, the fasting body weight is measured and set as 0w body weight. After 8 weeks of drug intervention, the body weight of mice in the Mod, SQL, and SQM increased (*p* < 0.05), with the Mod increasing most significantly (*p* < 0.01). At the end of 8 weeks, the body weight of mice in the drug group was compared with the Mod. There is a statistical difference (*p* < 0.05), among which SQH has the lowest weight (*p* < 0.01) (Table 3; Figure 7).

**TABLE 3:** Body weight in each group at different time points (*x* ± s, g,week).

Group	0w	2w	4w	6w	8w
Con	26.1 ± 0.6	27.1 ± 0.6	28.4 ± 0.6	29.2 ± 0.8	29.3 ± 1.4
Mod	25.2 ± 1.5	25.5 ± 1.3	26.1 ± 1.5	28.3 ± 1.7	31.7 ± 1.4^*^
SQL	24.2 ± 0.9	25.0 ± 0.4	25.2 ± 0.7	26.2 ± 0.9	27.7 ± 1.0^#^
SQM	24.3 ± 1.1	25.0 ± 1.1	25.5 ± 1.1	27.2 ± 1.0	28.3 ± 0.9^#^
SQH	24.1 ± 0.9	24.5 ± 0.5	25.5 ± 0.7	26.1 ± 0.7^#^	26.4 ± 0.7^##^
Rg	25.0 ± 0.8	25.3 ± 0.7	25.5 ± 0.6	26.3 ± 0.4^#^	27.1 ± 0.4^#^

Compared to Con.

*p < 0.05,^**^ *p* < 0.01; Compared to Mod.

^*#*^ *p* < 0.05;^##^ *p* < 0.01.

**FIGURE 8:**
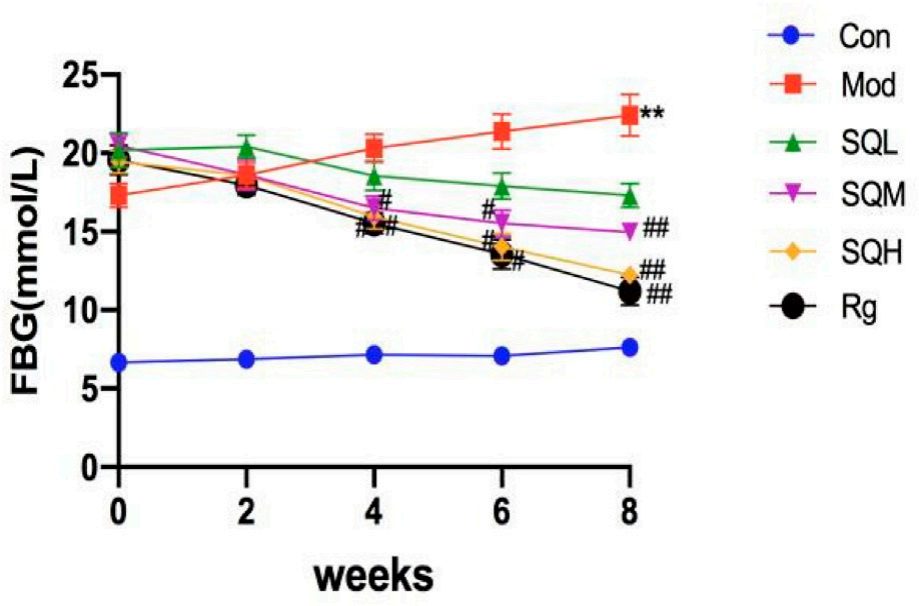
FBG of mice in each group at different time points.

FBG Compared with the Con, the FBG levels of mice in the Mod were increased from 0W to 8W (*p* < 0.01). Compared with the Mod, there was no significant difference in the FBG levels of mice in each drug intervention group before administration (*p* > 0.05). Starting from 4 weeks after administration, FBG in the SQM and SQH decreased compared with the Mod (*p* < 0.05). After 8 weeks of medication, FBG in the SQM and SQH was significantly lower than the Mod (*p* < 0.01) (Table 4; Figure 8).

**TABLE 4:** FBG in each group at different time points (*x* ± s,week).

Group	0w	2w	4w	6w	8w
Con	6.7 ± 0.4	6.9 ± 0.4	7.2 ± 0.3	7.1 ± 0.2	7.6 ± 0.3
Mod	17.3 ± 0.7^**^	18.6 ± 0.8^**^	20.3 ± 0.9^**^	21.4 ± 1.1^**^	22.4 ± 1.3^**^
SQL	20.2 ± 1.1^**^	20.4 ± 0.7^**^	18.6 ± 0.9^**^	17.9 ± 0.8^**^	17.3 ± 0.7^**^
SQM	20.5 ± 0.7^**^	18.6 ± 1.0^**^	16.5 ± 0.7^**#^	15.5 ± 0.8^**#^	15.0 ± 0.6^**##^
SQH	19.5 ± 0.7^**^	18.6 ± 0.5^**^	15.9 ± 0.8^**#^	14.0 ± 0.8^**#^	12.2 ± 0.5^**##^
Rg	19.6 ± 0.9^**^	18.0 ± 0.6^**^	15.5 ± 0.6^**#^	13.6 ± 0.9^**#^	11.2 ± 0.9^**##^

Compared to Con.

*p* < 0.05,^**^ *p* < 0.01; Compared to Mod.

*p* < 0.05;^##^ *p* < 0.01.

#### 3.2.3 SQP improves islet cell function

Compared to Con, the blood glucose levels in Mod were significantly elevated at all time points ranging from 0 to 120 min during OGTT (*p* < 0.01). Notably, the most pronounced increase in blood glucose levels in the Mod group occurred at 30 min post-glucose administration (*p* < 0.05). Subsequently, at the 60-min mark post-administration, a declining trend in blood glucose levels was observed across all drug intervention groups. Specifically, statistically significant differences were noted between SQM, SQH, and Mod (*p* < 0.01). Furthermore, after 120 min of glucose administration, the blood sugar levels of SQM, SQH, and Rg, statistically significant differences were observed (Table 5, *p* < 0.05).

**TABLE 5:** Effect of SQP on glucose tolerance (*x* ± s).

Group	Glucose (mmol/L)			
	0min	30min	60min	120min
Con	7.6 ± 0.4	11.8 ± 0.8	9.6 ± 0.8	6.3 ± 0.5
Mod	22.9 ± 1.3^**^	30.0 ± 1.2^**^	23.8 ± 3.3^**^	20.2 ± 1.4^**^
SQL	17.6 ± 0.7^*,#^	23.1 ± 2.1^*,#^	18.7 ± 3.2^*^	17.7 ± 5.5^*^
SQM	14.9 ± 0.6^*,#^	17.8 ± 0.8^*##^	13.2 ± 1.6^#^	10.5 ± 1.1^#^
SQH	11.9 ± 0.5^*,#^	15.7 ± 0.2^##^	12.6 ± 1.2^#^	9.5 ± 0.2^#^
Rg	11.4 ± 1.0^*,#^	15.8 ± 1.1^##^	12.1 ± 0.9^#^	10.0 ± 0.5^#^

Compared to Con.

*p* < 0.05,^**^ *p* < 0.01; Compared to Mod.

*p* < 0.05;^##^ *p* < 0.01.

**FIGURE 9:**
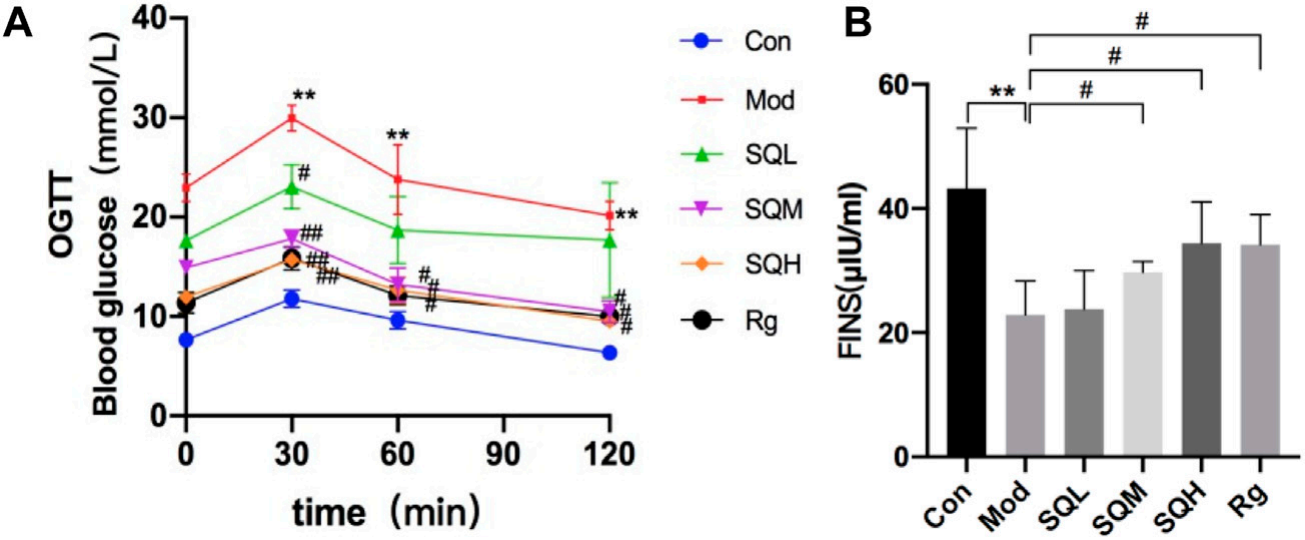
Effect of SQP on OGTT and FINS in mice. **(A)** OGTT in mice **(B)** FINS in mice. (Compared with Con, **p < 0.01; Compared with Mod, ^#^ *p* < 0.05).

FINS Compared with Con, the FINS level in the Mod was significantly lower (*p* < 0.01). Compared with Mod, the FINS levels of SQM and SQH increased (*p* < 0.05), and there was no statistical difference between SQL and Mod (*p* > 0.05) (Table 6; Figure 9A, B).

**TABLE 6:** Effects of SQP on FINS, HOMA-β, and ISI (*x* ± s).

Group	FINS (μIU/mL)	HOMA-β
Con	43.25 ± 9.22	208.64 ± 40.43
Mod	22.87 ± 5.19^**^	23.39 ± 4.05^**^
SQL	22.12 ± 6.25	31.45 ± 9.42
SQM	29.72 ± 1.65^#^	52.14 ± 4.25^#^
SQH	34.43 ± 6.34^#^	82.19 ± 17.49^#^
Rg	34.15 ± 4.64^#^	87.57 ± 12.59^#^

Compared to Con.

*p* < 0.05,^**^ *p* < 0.01; Compared to Mod.

*p* < 0.05.

**FIGURE 10:**
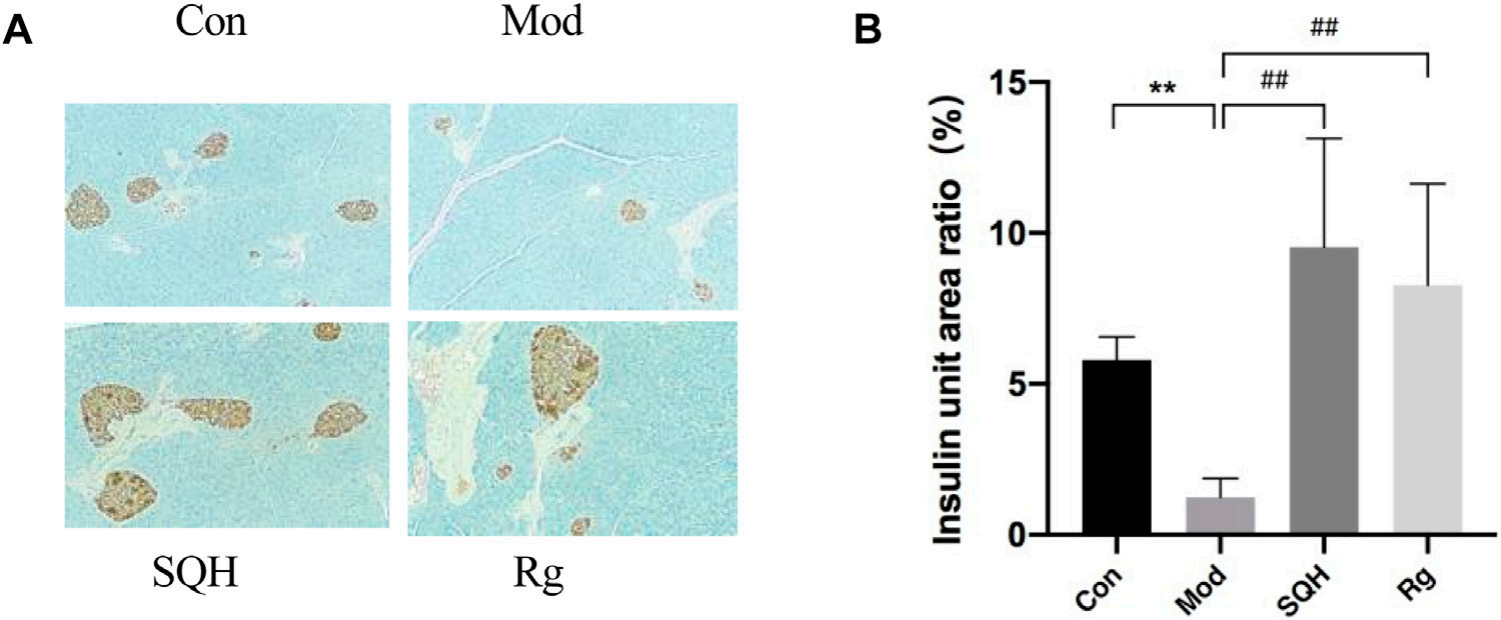
Effects of insulin secretion levels in pancreatic tissue of mice (400×) **(A)** Histochemical staining results of insulin in pancreatic tissue of mice under high magnification **(B)** Statistics of insulin levels per unit area of pancreatic tissue in mice. (Compared with Con, **p < 0.01; Compared with Mod, ^##^ *p* < 0.01) A.

Insulin immunohistochemical staining Con, Mod, SQH (according to the results of mice fasting insulin levels) and Rg mice pancreatic tissue were selected for insulin immunohistochemical staining. The results showed that under high magnification, Con insulin secretion granules were of equal size, and Mod was smaller. Insulin secretory granules decreased compared with Con (*p* < 0.01). SQH insulin secretion granules became larger, although the sizes varied, but the proportion of insulin volume per unit area was larger than that of the Mod (*p* < 0.01). The size of Rg insulin secretion granules was unequal, and it was also larger than that of Mod (*p* < 0.01). Apply ImageJ software to analyze statistical results (Figures 10A, B).

**FIGURE 11:**
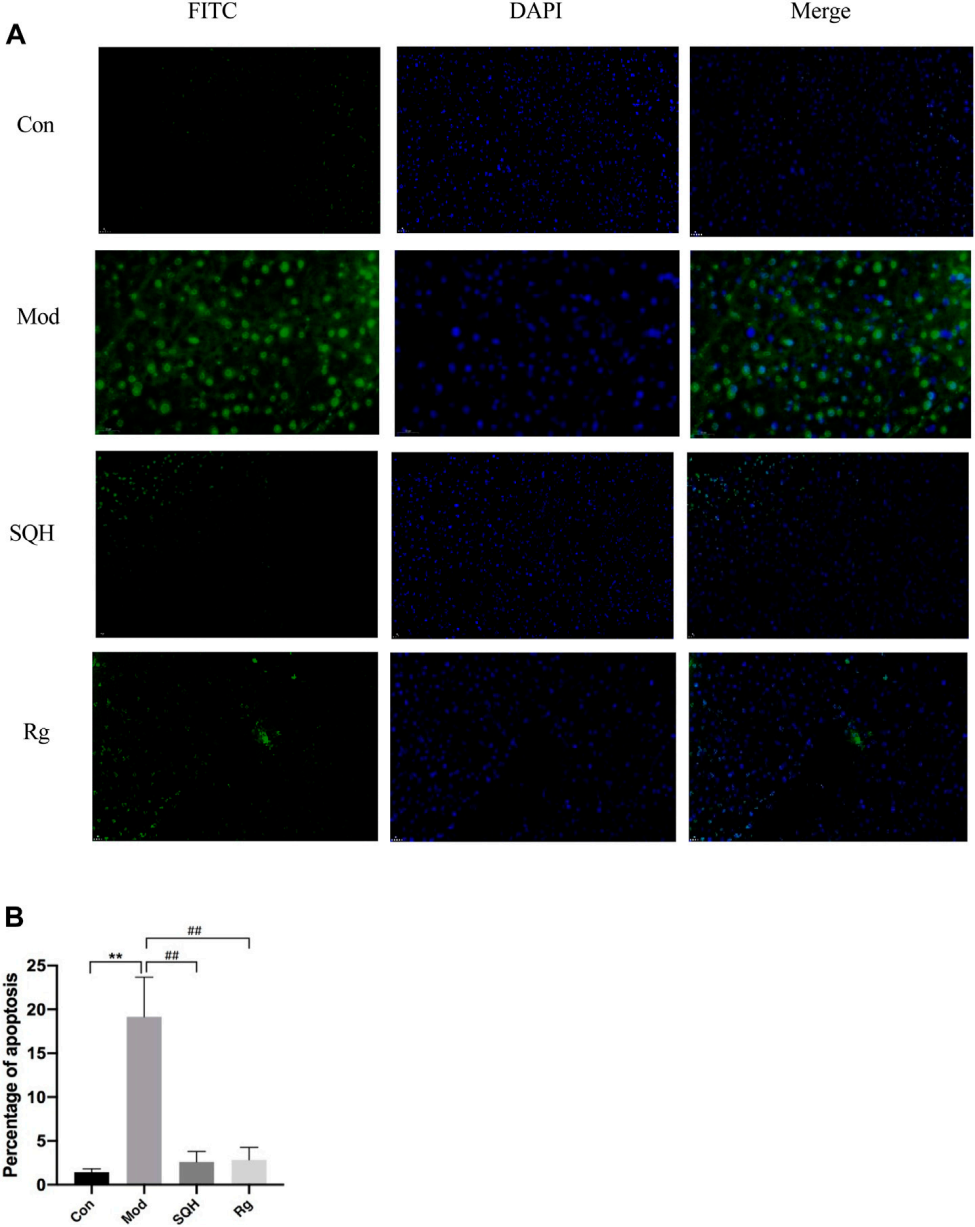
Apoptosis rate of pancreatic β cells in mice (400×) **(A)** Staining results of pancreatic β-cells in mice under a fluorescence microscope **(B)** Statistics of apoptosis of pancreatic β-cells in mice. (Compared with Con, **p < 0.01; Compared with Mod, ^##^ *p* < 0.01).

Pancreatic β-cells apoptosis The results of tunel apoptosis of pancreatic islet β-cells showed that compared with Con, the apoptosis rate of islet β-cells in Mod was significantly increased (*p* < 0.01). Compared with Mod, the apoptosis rates of pancreatic islet β cells in SQH and Rg were significantly reduced (*p* < 0.01). Apply ImageJ software to analyze statistical results (Figures 11A, B).

#### 3.2.4 SQP improves lipid metabolism, protects mitochondrial morphology, and reduces oxidative stress levels

**FIGURE 12:**
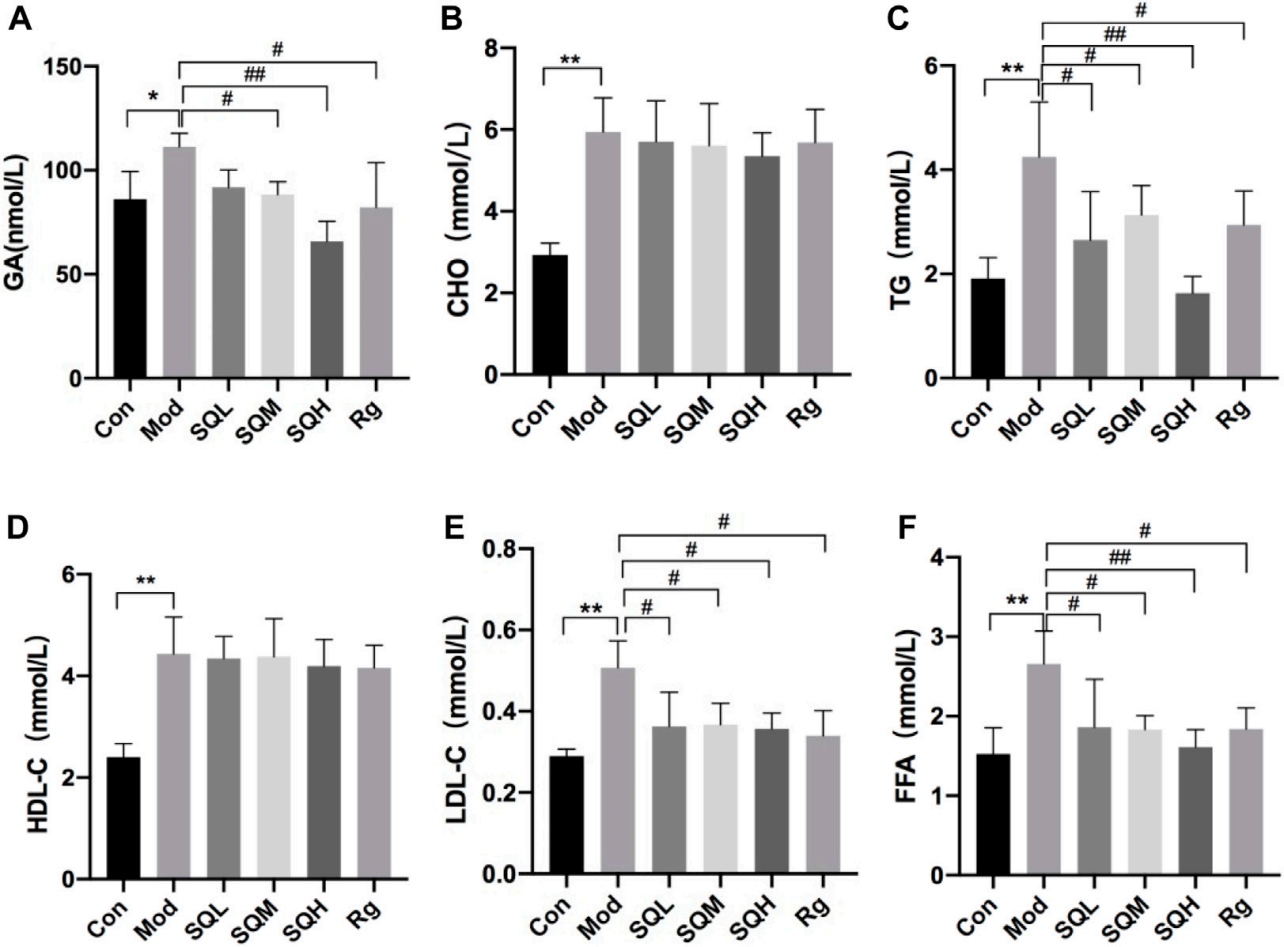
GA, CHO, TG, HDL-C, LDL-C and FFA levels of mice in each group **(A)** GA in mice; **(B–E)** Four blood lipids in mice; **(F)** FFA in mice. (Compared with Con, *p < 0.05, **p < 0.01; Compared with Mod, ^#^ *p* < 0.05; ^##^ *p* < 0.01).

GA Compared with the Con, the GA level in the Mod increased (*p* < 0.05); compared with the Mod, the GA level in the SQM, SQH and Rg decreased (*p* < 0.05 or *p* < 0.01). There was no statistically significant difference in GA between medication intervention groups (*p* > 0.05). Four items of blood lipids Compared with the Con, the levels of TG and LDL in the Mod increased (*p* < 0.01). Compared with the Mod, the levels of TG and LDL in each dose group of SQP and the Rg were reduced (*p* < 0.05 or *p* < 0.01). There was no statistically significant difference between the CHO and HDL medication intervention groups and the Mod (*p* > 0.05)。FFA Compared with the Con, the FFA level in the Mod increased (*p* < 0.01). Compared with the Mod, the FFA levels in each dose group of SQP and the Rg were reduced (*p* < 0.05 or *p* < 0.01), and the GA differences between each medication intervention group were no statistical significance (*p* > 0.05) (Table7; Figures 12A–F).

**TABLE 7:** GA, four blood lipids, and free fatty acid levels in each group (*x* ± s, mmol/L).

Group	GA (nmol/L)	CHO	TG	LDL-C	HDL-C	FFA
Con	86.06 ± 12.73	2.93 ± 0.27	1.91 ± 0.38	0.29 ± 0.02	2.40 ± 0.25	1.53 ± 0.31
Mod	111.31 ± 6.21^*^	5.94 ± 0.79^**^	4.25 ± 1.01^**^	0.51 ± 0.06^*^	4.43 ± 0.69^**^	2.49 ± 0.53^**^
SQL	91.84 ± 7.91	5.70 ± 0.95	2.82 ± 1.66^#^	0.38 ± 0.10^#^	4.34 ± 0.41	1.86 ± 0.58^#^
SQM	88.18 ± 5.87^#^	5.61 ± 0.98	3.13 ± 0.54^#^	0.37 ± 0.05^#^	4.38 ± 0.71	1.83 ± 0.17^#^
SQH	65.79 ± 9.16^##^	5.35 ± 0.54	1.63 ± 0.31^##^	0.36 ± 0.04^#^	4.19 ± 0.50	1.61 ± 0.21^##^
Rg	88.23 ± 20.52^#^	5.68 ± 0.77	2.94 ± 0.62^#^	0.34 ± 0.06^#^	4.16 ± 0.43	1.84 ± 0.25^#^

Compared to Con.

*p* < 0.05,^**^ *p* < 0.01; Compared to Mod.

*p* < 0.05.

**FIGURE 13:**
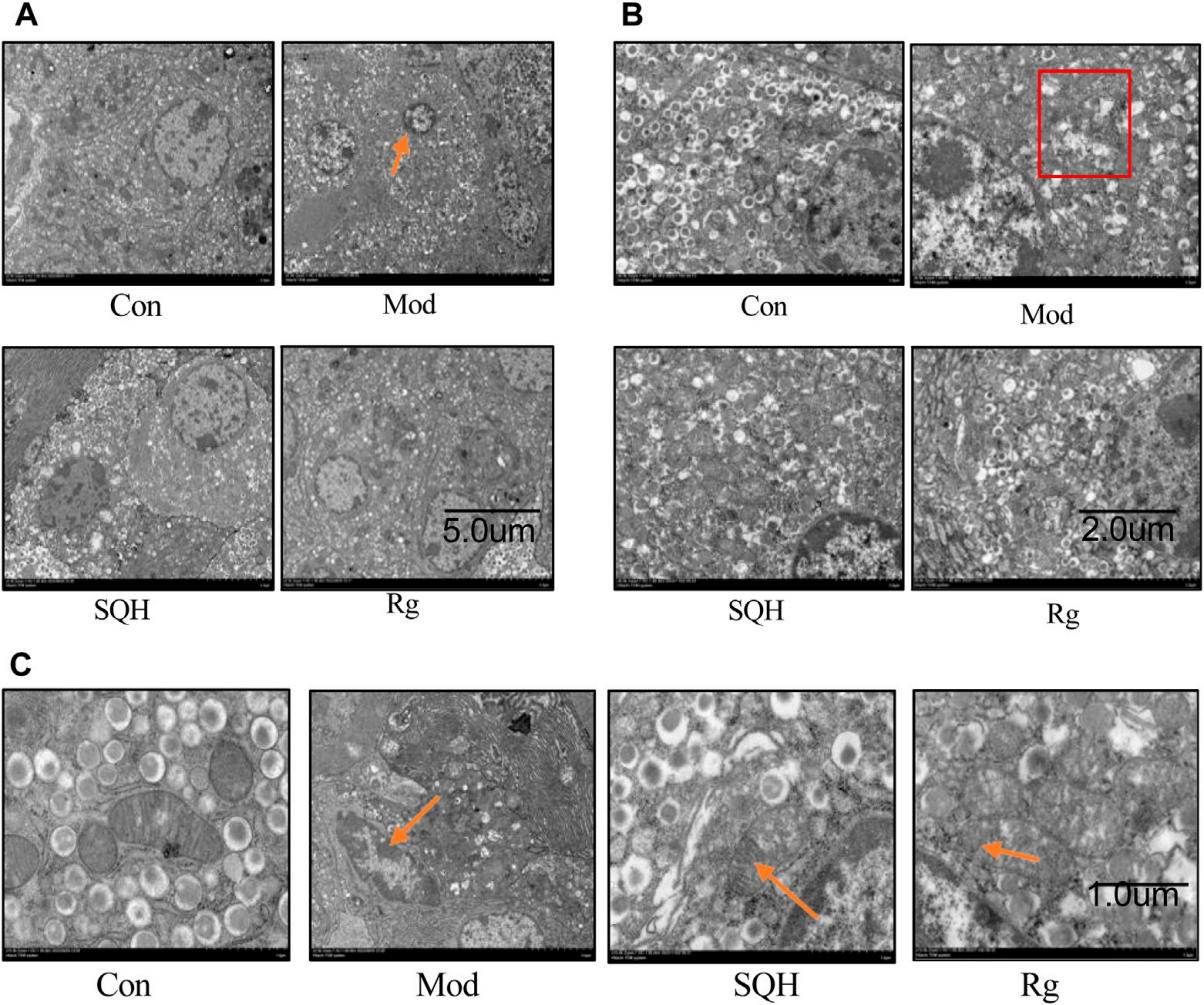
Transmission electron microscopy observation of the ultrastructure of pancreatic β cells. **(A)** Nucleus structure of pancreatic islet β cells **(B)** Cytoplasmic granule structure of pancreatic islet β cells **(C)** Mitochondrial structure of pancreatic islet β cells (The arrows in **(A)** point to the pyknotic nuclei of islet β cells. The boxes in **(B)** indicate reduced cytoplasmic granules. The arrows in the Mod in **(C)** point to mitochondrial cristae with broken morphology, and the arrows in the SQH and Rg point to the presence of normal mitochondrial cristae morphology.

The transmission electron microscopy results of the ultrastructural observation of the mitochondria of pancreatic islet β cells showed that the nucleus of Con islet β cells was large and round or oval in shape. There were a large number of secretory granules in the cytoplasm, most of which were spherical, with membranes on the outside and inside the granules. Cores with different electron densities are mostly round, with a large gap between the membrane and the core, bright and clear. Mitochondria have regular shapes and clear structures. Compared with Con, the Mod cells nucleus shrunk and showed an irregular shape, the number of cytoplasmic granules was reduced and unevenly distributed, the inner core shape of the granules was irregular, and the granules were not clear. The mitochondria were shrunken, the mitochondrial cristae were broken, and they gathered toward the base. Compared with Mod, the nuclei of SQH and Rg cells are enlarged, the nuclei are round, the number of cytoplasmic granules is increased, and the shape of the inner core of the granules is slightly regular. The mitochondria are regular in shape, and mitochondrial cristae are present (Figures 13A–C).

**FIGURE 14:**
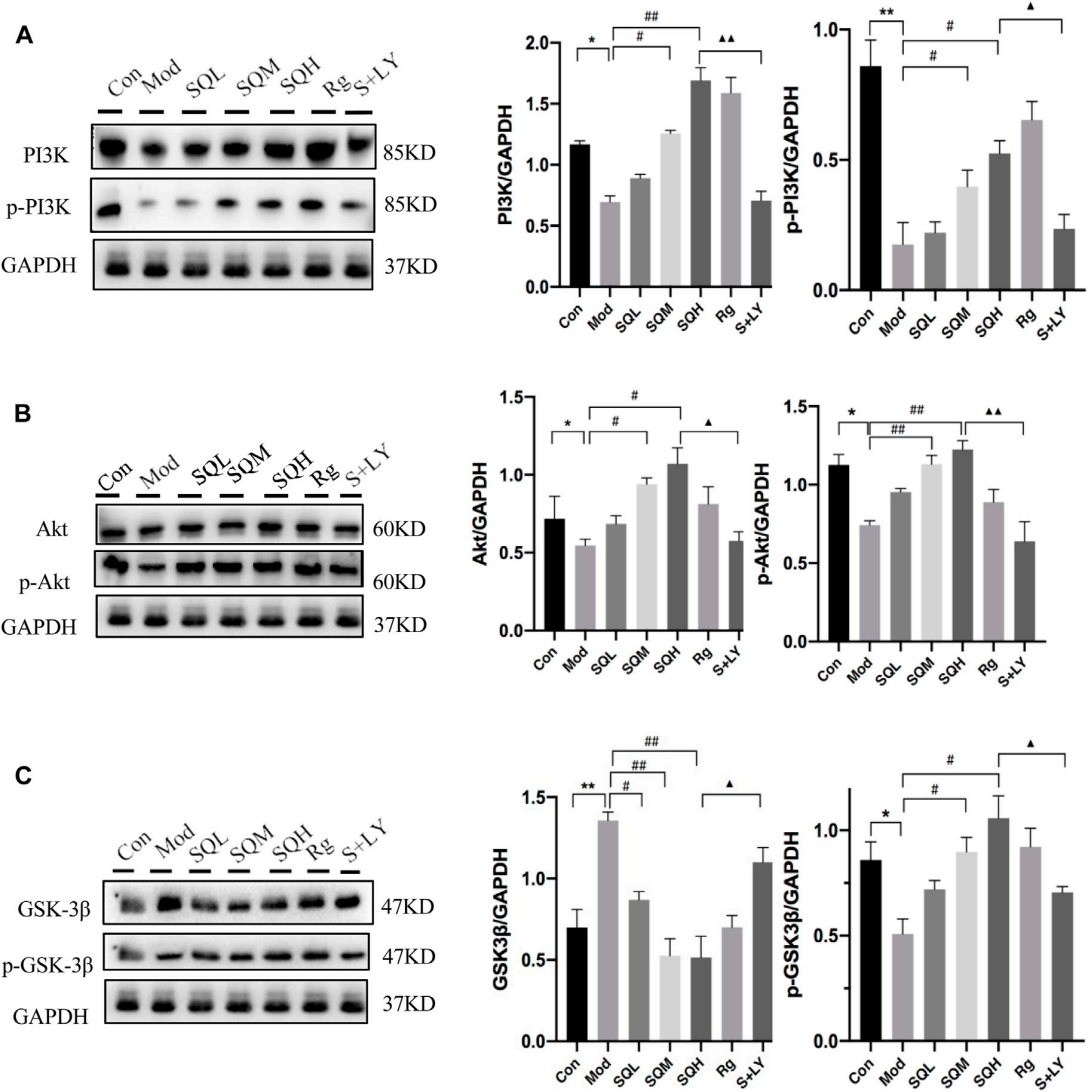
The expression of PI3K-Akt-GSK-3β signaling axis protein. **(A)** The expression of PI3K/p-PI3K **(B)** The expression of Akt/p-Akt **(C)** The expression of GSK-3β/p- GSK-3β [(Compared with Con, *p < 0.05, **p < 0.01; Compared with Mod, ^#^ *p* < 0.05, ^##^ *p* < 0.01; Compared with SQH, ▲*p* < 0.05, ▲▲*p* < 0.01)].

The expression of PI3K-Akt-GSK-3β signaling axis protein WB results show that compared with Con, the protein expression of PI3K/p-PI3K, Akt/p-Akt and p-GSK-3β in Mod decreased (*p* < 0.05 or *p* < 0.01), and the expression of GSK-3β increased (*p* < 0.01). Compared with Mod, the protein expressions of PI3K/p-PI3K, Akt/p-Akt and p-GSK-3β were increased in SQM, SQH and Rg (*p* < 0.05 or *p* < 0.01). Compared with SQH, the PI3K blocker group (LY294002, hereinafter referred to as the LY group) had reduced protein expressions of PI3K/p-PI3K, Akt/p-Akt and p-GSK-3β (*p* < 0.05 or *p* < 0.01), and increased GSK- 3β expression (*p* < 0.05) (Figures 14A–C).

## 4 Discussion

Given the multifaceted complications associated with Subclinical Diabetic Mellitus (SDM), including atypical symptoms, a high risk of hypoglycemia, and suboptimal patient self-management, standardized management of SDM constitutes a crucial component of clinical intervention (Lin et al., 2016; Yu et al., 2020; Yu et al., 2022). Particularly noteworthy is the frequent coexistence of SDM with various chronic conditions, underscoring the importance of actively pursuing comprehensive diagnostic and therapeutic measures.

As a component of alternative medicine, Chinese herbal medicine has been widely clinically accepted because of its safety and multi-target effects (Xu et al., 2015; Wang et al., 2018). According to the principles of traditional Chinese medicine, SDM is mainly characterized by deficiency, among which the “pi” and “shen” are the key organs (Chen and He, 2020). Shenqi pills have always been used as a basic prescription to nourish the “shen” and replenish “qi”. An improved version of Shenqi Pills (SQP) omits the pungent aconite to reduce potential damage to body fluids and enhance “pi” function. Noteworthy ingredients include codonopsis pilosula and astragalus membranaceus to nourish qi, trigonella foenum-graecum to nourish the “shen” and warm “yang qi”, purslane to strengthen the “pi” and remove dampness. Clinical studies have confirmed that Shenqi Pills can improve the symptoms of polydipsia and polyuria and maintain blood sugar stability in SDM patients. However, its precise mechanism of action remains elusive. Therefore, this study is dedicated to identifying target genes related to SQP and SDM through gene database exploration, and conducting GO enrichment analysis through network review to elucidate its putative mechanism. Furthermore, we designed in experiments to confirm the feasibility of the theoretical framework.

Mass spectrometry analysis of the SQP formulation revealed Catalpol as the main component, belonging to the iridoid glycosides class. Iridoid glycosides are known to enhance lipid metabolism, alleviate insulin resistance, and stimulate insulin secretion, thereby exerting a hypoglycemic effect (Bhattamisra et al., 2019; Bhattamisra et al., 2021). This pharmacological insight furnishes experimental validation for subsequent SQP investigations. In this investigation, a comprehensive screening process involving various databases and subsequent Venn analysis identified 166 potential targets of SQP pertinent to SDM treatment. Visualization of these findings was facilitated through PPI diagrams. To illuminate the multifaceted mechanisms underlying SQP’s efficacy in SDM management, we conducted GO enrichment analysis on intersecting target genes. The outcomes revealed a predominant enrichment of target genes in key biological processes, notably oxidative stress response, inflammatory response, and lipopolysaccharide responsiveness. Oxidative stress stands as a significant risk factor in SDM pathology, capable of activating multiple pathways leading to cellular damage. Notably, the excessive generation of mitochondrial superoxide anions induced by elevated sugar and fat levels serves as a primary trigger for cellular injury (Andreas et al., 2019; Han et al., 2021; Pruett et al., 2022). The pivotal role of oxidative stress-induced mitochondrial dysfunction in SDM pathogenesis has garnered widespread attention (Yin and Ding, 2013; Andrieux et al., 2021; Sulkshane et al., 2021). Given mitochondria’s central role in cellular oxidative stress, we identified mitochondria-related genes via the MitoCarta3.0 database. Subsequently, we intersected these genes with the co-regulated targets of SDM and SQP, leveraging the GeneMANIA database to discern the top five functions of the resultant intersection genes: mitochondrial outer membrane and embedded mitochondrial membrane proteins, apoptosis signaling pathway involvement, positive regulation of apoptosis signaling pathway, and regulation of mitochondrial membrane permeability. These findings suggest that SQP’s therapeutic action in SDM may encompass enhancement of biological processes such as oxidative stress and inflammatory response, with pertinent mechanisms potentially involving alterations in mitochondrial morphological structure and the expression of apoptosis pathway proteins.

To further corroborate our preceding findings, we devised *in vivo* experiments aimed at elucidating the impact of oxidative stress on glucose and lipid metabolism dysregulation in mice, alongside associated pathways. Employing a combination of high-sugar and high-fat diet alongside streptozotocin (STZ) induction, we established an SDM model in C57BL/6 mice. Given recent research correlating cellular aging with metabolic reprogramming pathways encompassing autophagy, oxidative stress, epigenetic modifications, and chronic inflammation, we procured 6-week-old mice to undergo a transition from adaptive feeding to a high-sugar and high-fat regimen spanning 9 weeks (Finkel and Oxidants, 2000; Klionsky et al., 2021; Li et al., 2023; Maldonado et al., 2023). Subsequently, SQP intervention was administered for 8 weeks, culminating in mice aged 23 weeks at the study’s conclusion. The utilization of a high-sugar and high-fat diet aimed to foster an internal milieu conducive to oxidative stress and chronic inflammation, thus inducing an SDM mouse model (Ferrucci and Fabbri, 2018). Post-intervention assessments revealed a controlled weight gain trajectory in mice receiving SQP. Serum analyses indicated reduced fasting blood sugar and serum albumin levels in the TCM group compared to the control, indicative of SQP’s potential to ameliorate weight gain induced by the high-sugar and high-fat diet while mitigating its impact on blood sugar levels (Li et al., 2019; Lytrivi et al., 2020a; Geidl-Flueck et al., 2021). Chronic inflammatory responses are known to precipitate dyslipidemia and elevated FFA levels, complicating glucose metabolism and insulin resistance. Notably, SQP intervention led to significant reductions in serum TG, LDL-C, and FFA levels, suggesting a potential role in attenuating insulin resistance by dampening inflammatory responses. Moreover, the high-sugar and high-fat diet-induced oxidative stress milieu impacts pancreatic β-cells apoptosis. OGTT and fasting insulin FINS measurements post-SQP intervention revealed improved pancreatic islet function in SDM mice. Immunohistochemical analysis of insulin secretion demonstrated enhanced pancreatic β-cells functionality following SQP administration. Additionally, tunel staining highlighted a reduction in apoptotic β cells under high-sugar and high-fat conditions, further underscoring SQP’s potential to mitigate β-cells apoptosis in such environments.

Regarding the verification of the mechanism of inflammation and oxidative stress on β-cells apoptosis, we used electron microscopy to observe the ultrastructure of β-cells (Lytrivi et al., 2020b). The results showed that SQP can protect the integrity of β-cells mitochondrial structure and morphology, and protect the existence of mitochondrial cristae morphology, confirming the above theoretical results of intersection target genes in theory. Glycogen synthase kinase-3 reduces glycogen synthesis by inhibiting glycogen synthase in the insulin signaling pathway and leads to insulin resistance (Akhtar and Sah, 2020). GSK-3β is a multifunctional serine/threonine kinase that belongs to the glycogen synthase kinase subfamily and is involved in inflammation, endoplasmic reticulum stress, mitochondrial dysfunction, and apoptosis pathways. Considered a negative regulator of β-cells mass in type 2 diabetes, p-GSK exerts anti-apoptotic effects. Both *in vivo* and *in vitro* experimental investigations have delineated the pivotal role of GSK-3 activity in governing mitochondrial function within skeletal muscle cells. Specifically, studies involving GSK-3 knockout (KO) have demonstrated a reduction in mitochondrial oxidative damage coupled with an augmentation of mitochondrial biogenesis (Wang et al., 2020; Cui et al., 2022; Cao et al., 2023). Its inactivation is usually caused by phosphorylation of substrates. GSK-3β in islet β cells is related to oxidative stress-induced apoptosis (Kim et al., 2010). The PI3K/Akt signaling pathway predominantly governs the phosphorylation of glycogen synthase kinase-3β (GSK-3β) at the serine nine site (Ser9). Upon activation, Akt acts as an upstream regulator of GSK-3β. Activation of Akt leads to its interaction with GSK-3β, resulting in the phosphorylation of the serine nine residue and subsequent inactivation of GSK-3β. This inactivation contributes to an anti-apoptotic effect (Qian et al., 2021; Nagao et al., 2022). Therefore, this study selected the PI3K/Akt/GSK-3β signaling pathway to investigate the impact of oxidative stress on pancreatic islet β cells. The findings indicate that a high-sugar and high-fat diet can elevate the expression of GSK-3β in pancreatic islet cells. However, intervention with SQP results in increased expression of p-GSK-3β, which inversely correlates with apoptosis, along with a reduction in GSK-3β expression. Furthermore, by enhancing the expression of upstream PI3K/Akt and employing a PI3K blocker, the study successfully blocked the aforementioned effects of SQP, confirming that SQP may mitigate oxidative stress-induced β-cell apoptosis via modulation of the PI3K/Akt/GSK-3β signaling pathway.

Hence, through a combination of network analysis and experimental validation, the study substantiates the efficacy of SQP in treating SDM by ameliorating oxidative stress responses. This therapeutic mechanism is postulated to operate via modulation of the PI3K/Akt/GSK-3β signaling pathway, coupled with the preservation of mitochondrial cristae morphology. Consequently, SQP attenuates the adverse effects of oxidative stress responses on pancreatic β-cell apoptosis induced by high-sugar and high-fat diets, thereby safeguarding pancreatic islet β cells.

## 5 Conclusion

Improving the internal environment of oxidative stress can help protect pancreatic β-cells damage caused by type 2 diabetes. This study predicts the effective mechanism and possible targets of SQP in treating SDM. *In vivo* studies, SQP demonstrates efficacy in ameliorating the internal environment characterized by oxidative stress through the stabilization of blood sugar levels and regulation of blood lipids. Consequently, this intervention contributes to the preservation of mitochondrial morphology. SQP exhibits the potential to mitigate β-cell apoptosis by modulating the PI3K/Akt/GSK-3β signaling pathway. These results provide empirical evidence for the application of SQP in diabetes management. This study combines network analysis and *in vivo* experimental validation. This study aims to strengthen the scientific basis for SQP application and promote the widespread application of traditional Chinese medicine in clinical practice.

## Data Availability

The data supporting the findings of this study are available within the article/Supplementary Material. The data of the study are openly available in GeneCards (https://www.genecards.org/), OMIM (https://omim.org/), PharmGkb (https://www.pharmgkb.org/), BATMAN-TCM (http://bionet.ncpsb.org.cn/batman-tcm/), Uniprot (https://www.uniprot.org/), MitoCarta3.0 (https://www.broadinstitute.org/mitocarta/mitocarta30-inventory-mammalian-mitochondrial-proteins-and-pathways) and GeneMANIA (http://genemania.org/) database.
