# Supplementary figures and images for: Network analysis combined with experimental assessment to explore the therapeutic mechanisms of New Shenqi Pills formula targeting mitochondria on senile diabetes mellitus
Source: Front Pharmacol. 2024 Jun 12;15:1339758. doi: 10.3389/fphar.2024.1339758 (PMC11211868; doi:10.3389/fphar.2024.1339758)

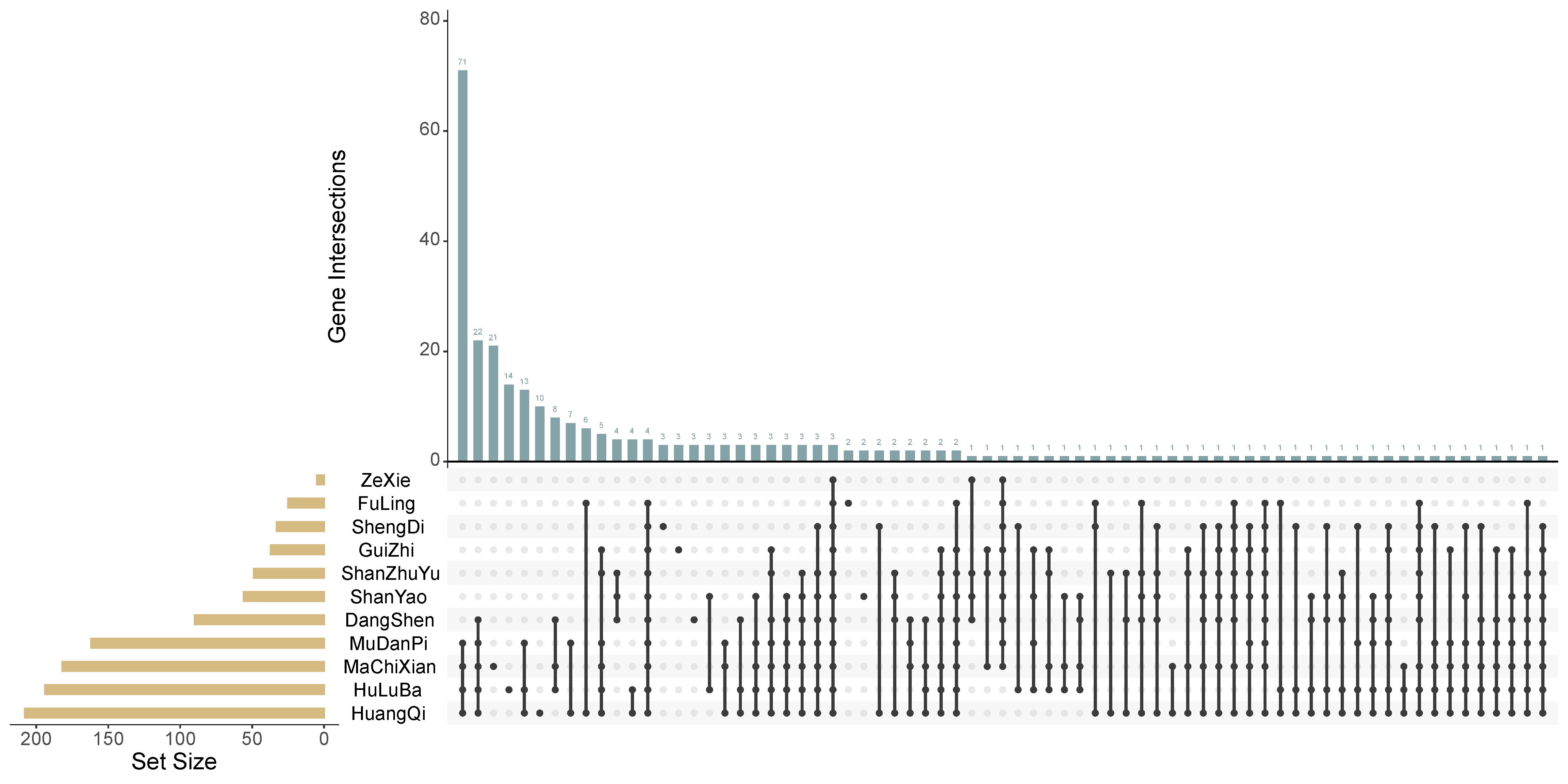

Supplement: Supplementary file 1 [file DataSheet1.ZIP › Appendix 1-Data of network analysis/drug/UpSet (1).png]

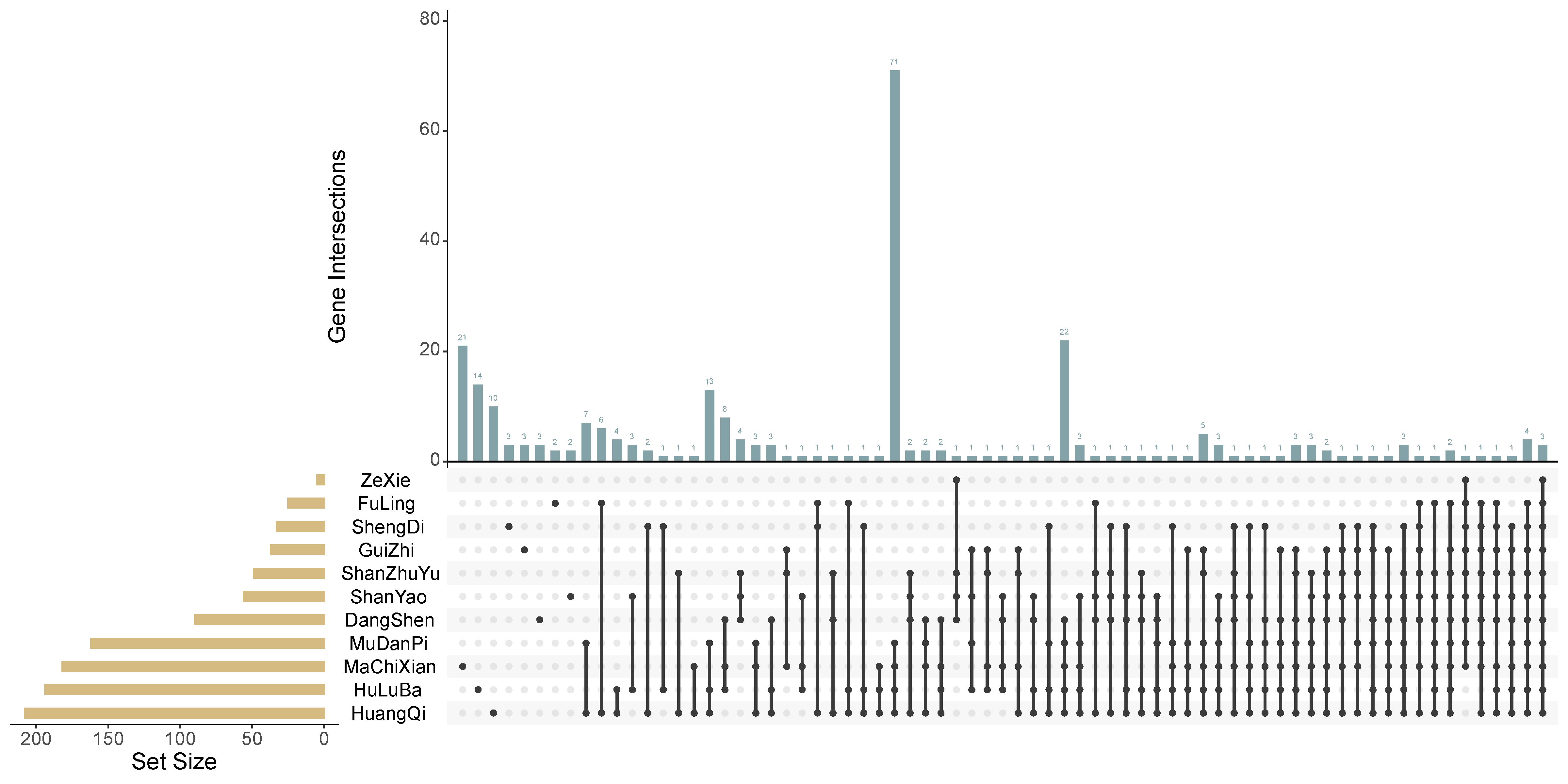

Supplement: Supplementary file 1 [file DataSheet1.ZIP › Appendix 1-Data of network analysis/drug/Upset.png]

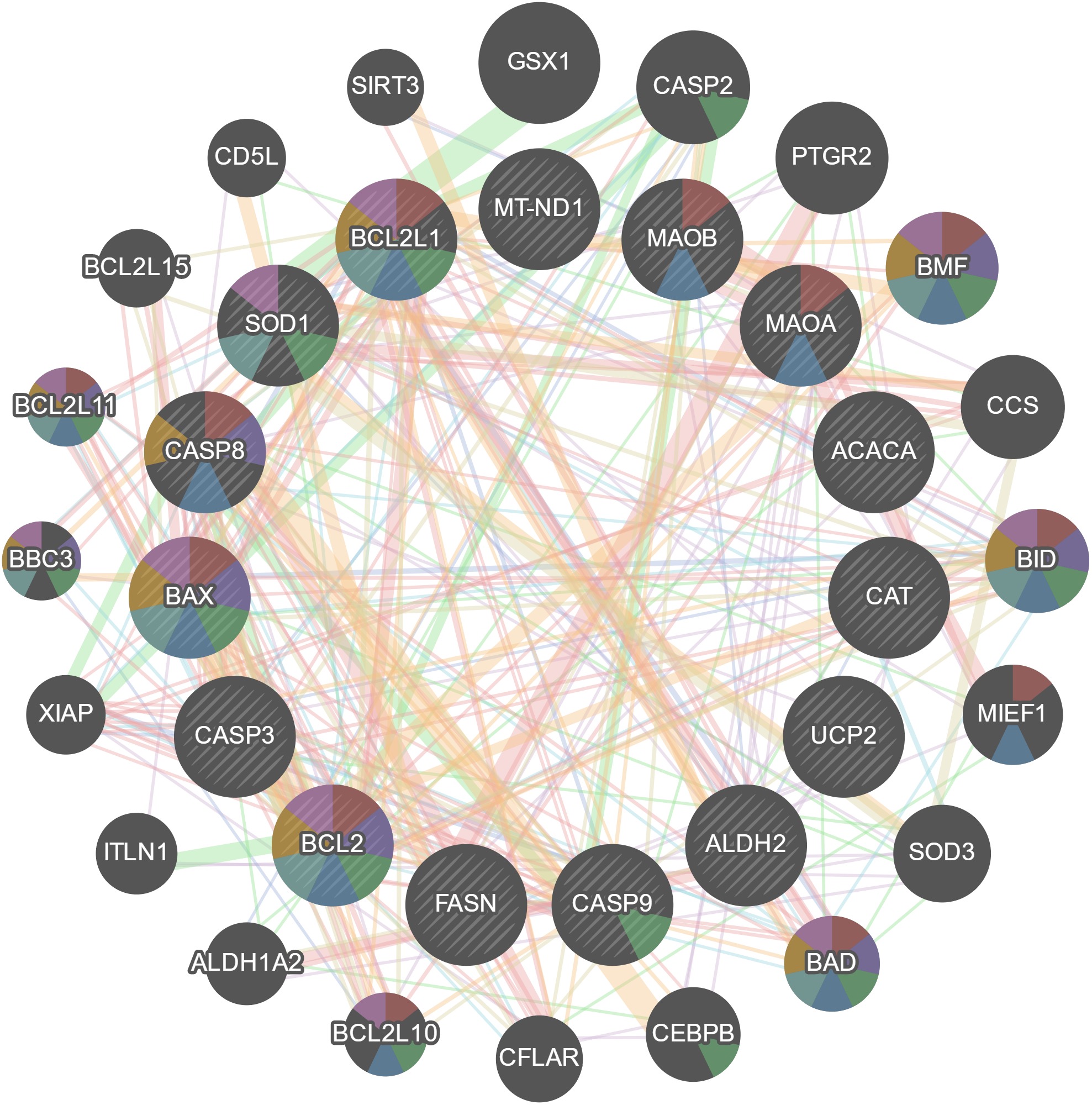

Supplement: Supplementary file 1 [file DataSheet1.ZIP › Appendix 1-Data of network analysis/geneMANIA/genemania-network.jpg]

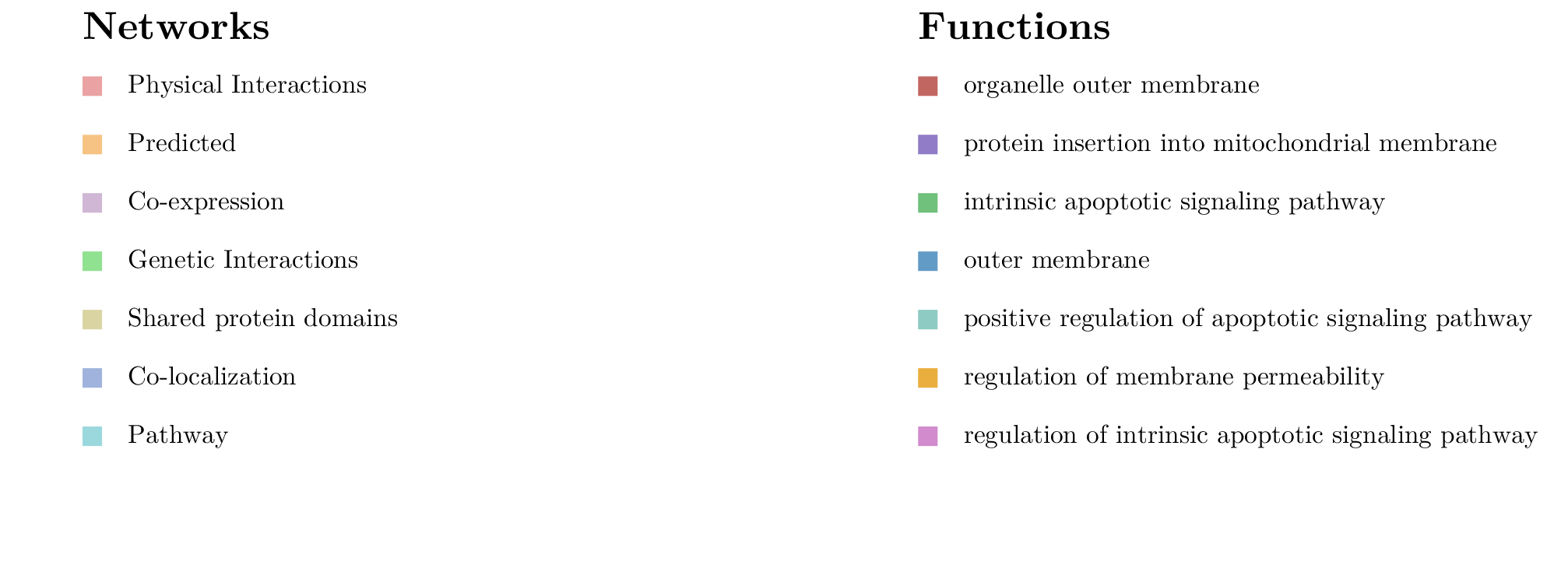

Supplement: Supplementary file 1 [file DataSheet1.ZIP › Appendix 1-Data of network analysis/geneMANIA/genemania-report-1.png]

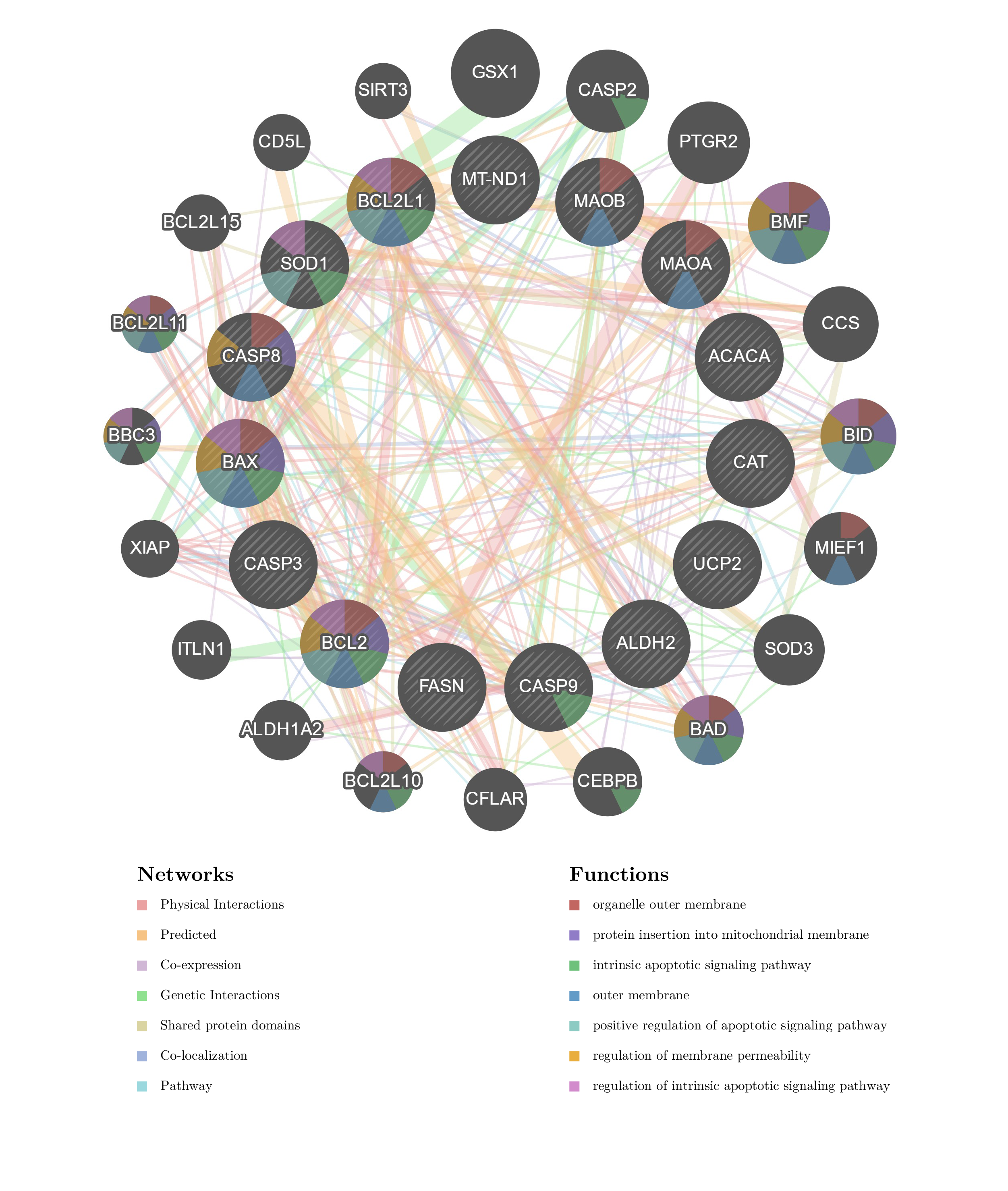

Supplement: Supplementary file 1 [file DataSheet1.ZIP › Appendix 1-Data of network analysis/geneMANIA/genemania.png]

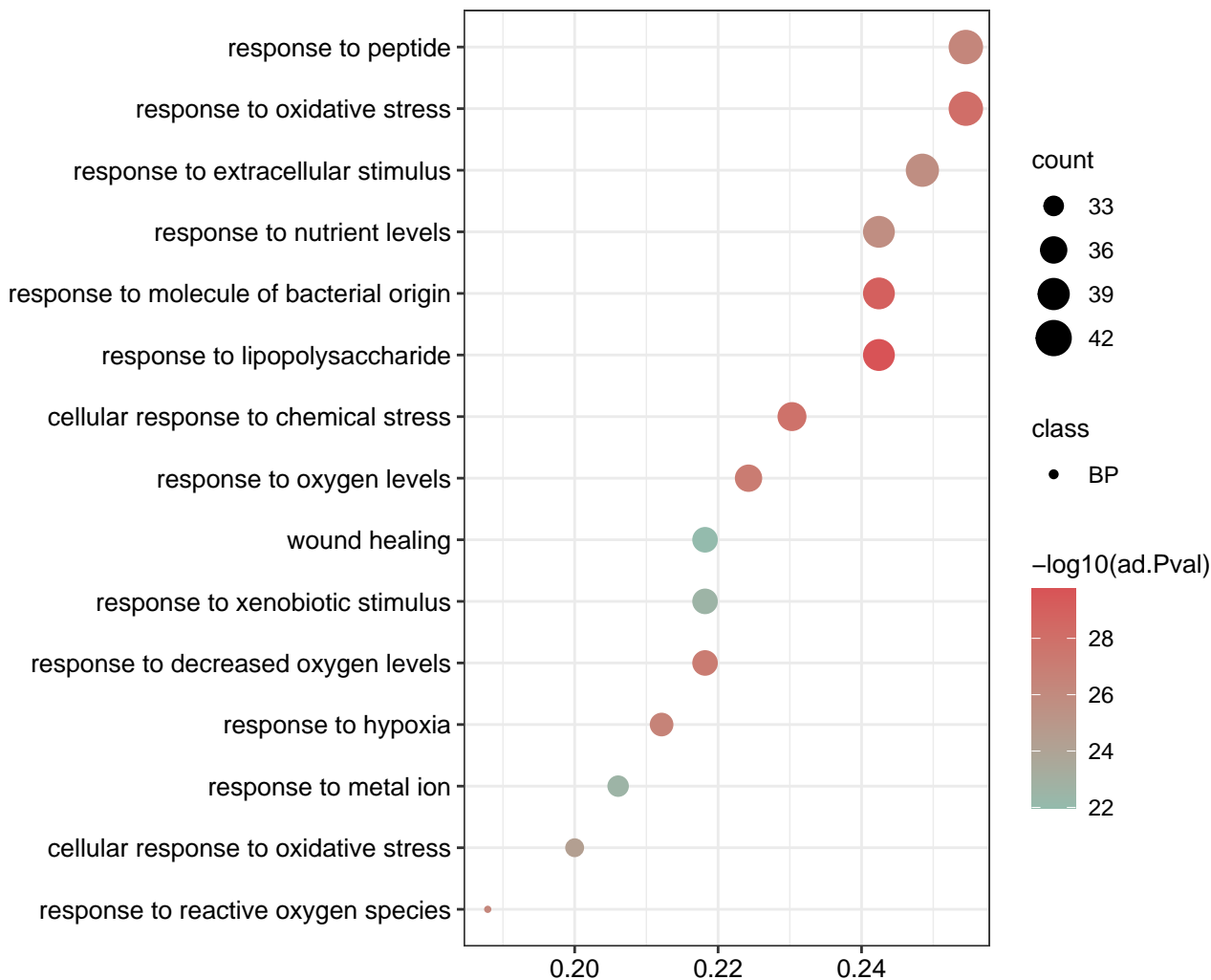

Supplement: Supplementary file 1 [file DataSheet1.ZIP › Appendix 1-Data of network analysis/GOandKEGG/GO-BP.pdf]

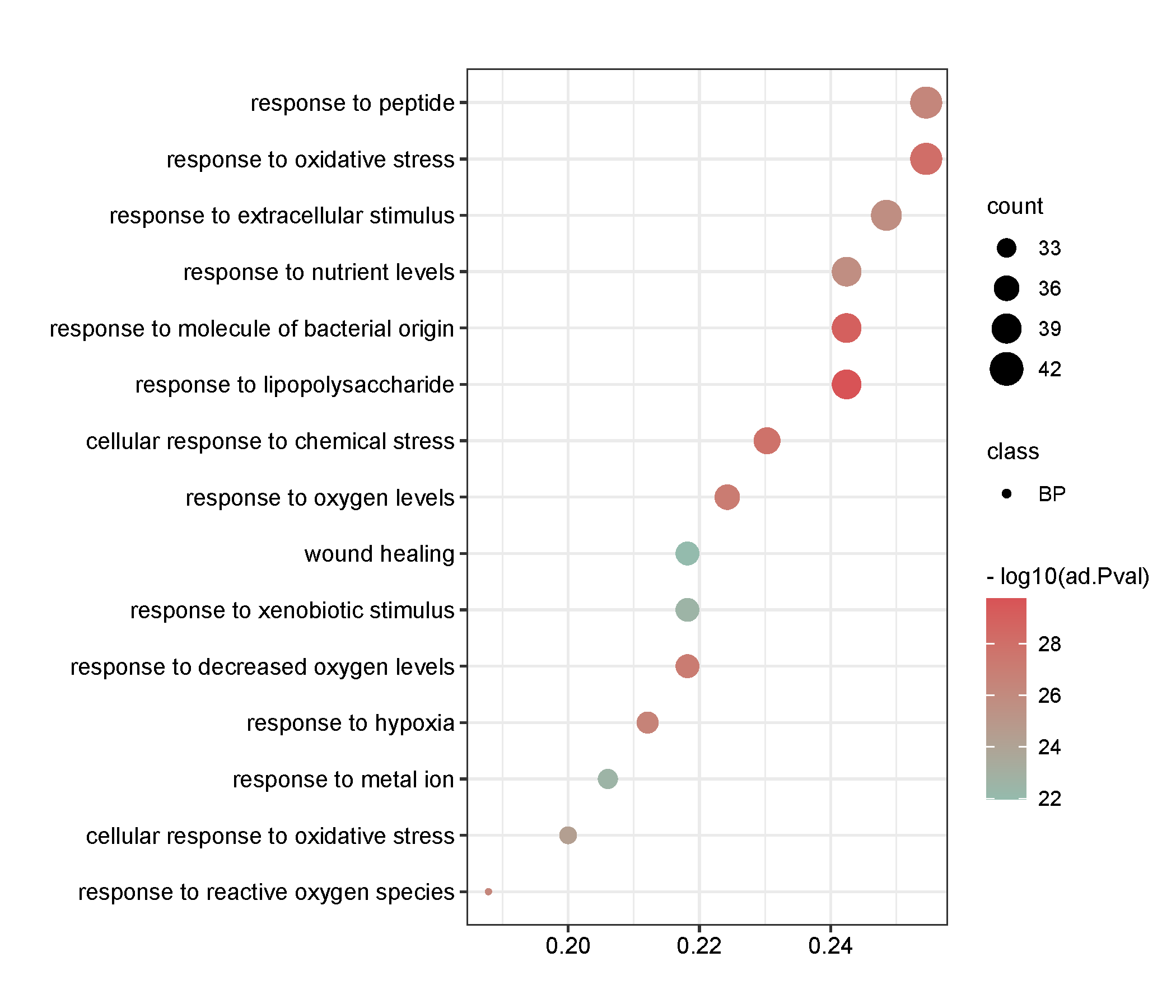

Supplement: Supplementary file 1 [file DataSheet1.ZIP › Appendix 1-Data of network analysis/GOandKEGG/GO-BP.png]
