# Supplementary material for: Network analysis combined with experimental assessment to explore the therapeutic mechanisms of New Shenqi Pills formula targeting mitochondria on senile diabetes mellitus
Source: Front Pharmacol. 2024 Jun 12;15:1339758. doi: 10.3389/fphar.2024.1339758 (PMC11211868; doi:10.3389/fphar.2024.1339758)
Supplement: Supplementary file 2 [file DataSheet2.docx]

Supplementary Material

# Supplementary Data

Table3 Body weight in each group at different time points（𝑥̅±s, g,week）

| Group | 0w | 2w | 4w | 6w | 8w |
| --- | --- | --- | --- | --- | --- |
| Con | 26.1±0.6 | 27.1±0.6 | 28.4±0.6 | 29.2±0.8 | 29.3±1.4 |
| Mod | 25.2±1.5 | 25.5±1.3 | 26.1±1.5 | 28.3±1.7 | 31.7±1.4^*^ |
| SQL | 24.2±0.9 | 25.0±0.4 | 25.2±0.7 | 26.2±0.9 | 27.7±1.0^#^ |
| SQM | 24.3±1.1 | 25.0±1.1 | 25.5±1.1 | 27.2±1.0 | 28.3±0.9^#^ |
| SQH | 24.1±0.9 | 24.5±0.5 | 25.5±0.7 | 26.1±0.7^#^ | 26.4±0.7^##^ |
| Rg | 25.0±0.8 | 25.3±0.7 | 25.5±0.6 | 26.3±0.4^#^ | 27.1±0.4^#^ |

Compared to Con, ^*^*P*<0.05，^**^*P*<0.01；Compared to Mod, ^#^*P*<0.05；^##^*P*<0.01

Table4 FBG in each group at different time points（𝑥̅±s,week）

| Group | 0w | 2w | 4w | 6w | 8w |
| --- | --- | --- | --- | --- | --- |
| Con | 6.7±0.4 | 6.9±0.4 | 7.2±0.3 | 7.1±0.2 | 7.6±0.3 |
| Mod | 17.3±0.7^**^ | 18.6±0.8^**^ | 20.3±0.9^**^ | 21.4±1.1^**^ | 22.4±1.3^**^ |
| SQL | 20.2±1.1^**^ | 20.4±0.7^**^ | 18.6±0.9^**^ | 17.9±0.8^**^ | 17.3±0.7^**^ |
| SQM | 20.5±0.7^**^ | 18.6±1.0^**^ | 16.5±0.7^**#^ | 15.5±0.8^**#^ | 15.0±0.6^**##^ |
| SQH | 19.5±0.7^**^ | 18.6±0.5^**^ | 15.9±0.8^**#^ | 14.0±0.8^**#^ | 12.2±0.5^**##^ |
| Rg | 19.6±0.9^**^ | 18.0±0.6^**^ | 15.5±0.6^**#^ | 13.6±0.9^**#^ | 11.2±0.9^**##^ |

Compared to Con, ^*^*P*<0.05，^**^*P*<0.01；Compared to Mod, ^#^*P*<0.05；^##^*P*<0.01

Table5 Effect of SQP on glucose tolerance（𝑥̅±s）

| Group | Glucose（mmol/L） | | | |
| --- | --- | --- | --- | --- |
|  | 0min | 30min | 60min | 120min |
| Con | 7.6±0.4 | 11.8±0.8 | 9.6±0.8 | 6.3±0.5 |
| Mod | 22.9±1.3^**^ | 30.0±1.2^**^ | 23.8±3.3^**^ | 20.2±1.4^**^ |
| SQL | 17.6±0.7^*#^ | 23.1±2.1^*#^ | 18.7±3.2^*^ | 17.7±5.5^*^ |
| SQM | 14.9±0.6^*#^ | 17.8±0.8^*##^ | 13.2±1.6^#^ | 10.5±1.1^#^ |
| SQH | 11.9±0.5^*#^ | 15.7±0.2^##^ | 12.6±1.2^#^ | 9.5±0.2^#^ |
| Rg | 11.4±1.0^*#^ | 15.8±1.1^##^ | 12.1±0.9^#^ | 10.0±0.5^#^ |

Compared to Con, ^*^*P*<0.05，^**^*P*<0.01；Compared to Mod, ^#^*P*<0.05；^##^*P*<0.01

Table6 Effects of SQP on FINS, HOMA-β, and ISI (𝑥̅±s）

| Group | FINS（μIU/ml） | HOMA-β |
| --- | --- | --- |
| Con | 43.25±9.22 | 208.64±40.43 |
| Mod | 22.87±5.19^**^ | 23.39±4.05^**^ |
| SQL | 22.12±6.25 | 31.45±9.42 |
| SQM | 29.72±1.65^#^ | 52.14±4.25^#^ |
| SQH | 34.43±6.34^#^ | 82.19±17.49^#^ |
| Rg | 34.15±4.64^#^ | 87.57±12.59^#^ |

Compared to Con, ^*^*P*<0.05，^**^*P*<0.01；Compared to Mod, ^#^*P*<0.05

Table7 GA, four blood lipids, and free fatty acid levels in each group（𝑥̅±s，mmol/L）

| Group | GA（nmol/L） | CHO | TG | LDL-C | HDL-C | FFA |
| --- | --- | --- | --- | --- | --- | --- |
| Con | 86.06±12.73 | 2.93±0.27 | 1.91±0.38 | 0.29±0.02 | 2.40±0.25 | 1.53±0.31 |
| Mod | 111.31±6.21^*^ | 5.94±0.79^**^ | 4.25±1.01^**^ | 0.51±0.06^*^ | 4.43±0.69^**^ | 2.49±0.53^**^ |
| SQL | 91.84±7.91 | 5.70±0.95 | 2.82±1.66^#^ | 0.38±0.10^#^ | 4.34±0.41 | 1.86±0.58^#^ |
| SQM | 88.18±5.87^#^ | 5.61±0.98 | 3.13±0.54^#^ | 0.37±0.05^#^ | 4.38±0.71 | 1.83±0.17^#^ |
| SQH | 65.79±9.16^##^ | 5.35±0.54 | 1.63±0.31^##^ | 0.36±0.04^#^ | 4.19±0.50 | 1.61±0.21^##^ |
| Rg | 88.23±20.52^#^ | 5.68±0.77 | 2.94±0.62^#^ | 0.34±0.06^#^ | 4.16±0.43 | 1.84±0.25^#^ |

Compared to Con, ^*^*P*<0.05，^**^*P*<0.01；Compared to Mod, ^#^*P*<0.05

**Please see Appendix 2 for specific data.**

# Supplementary Figures and Tables

For more information on Supplementary Material and for details on the different file types accepted, please see [here](https://www.frontiersin.org/guidelines/author-guidelines#supplementary-material).

## Supplementary Figures

**
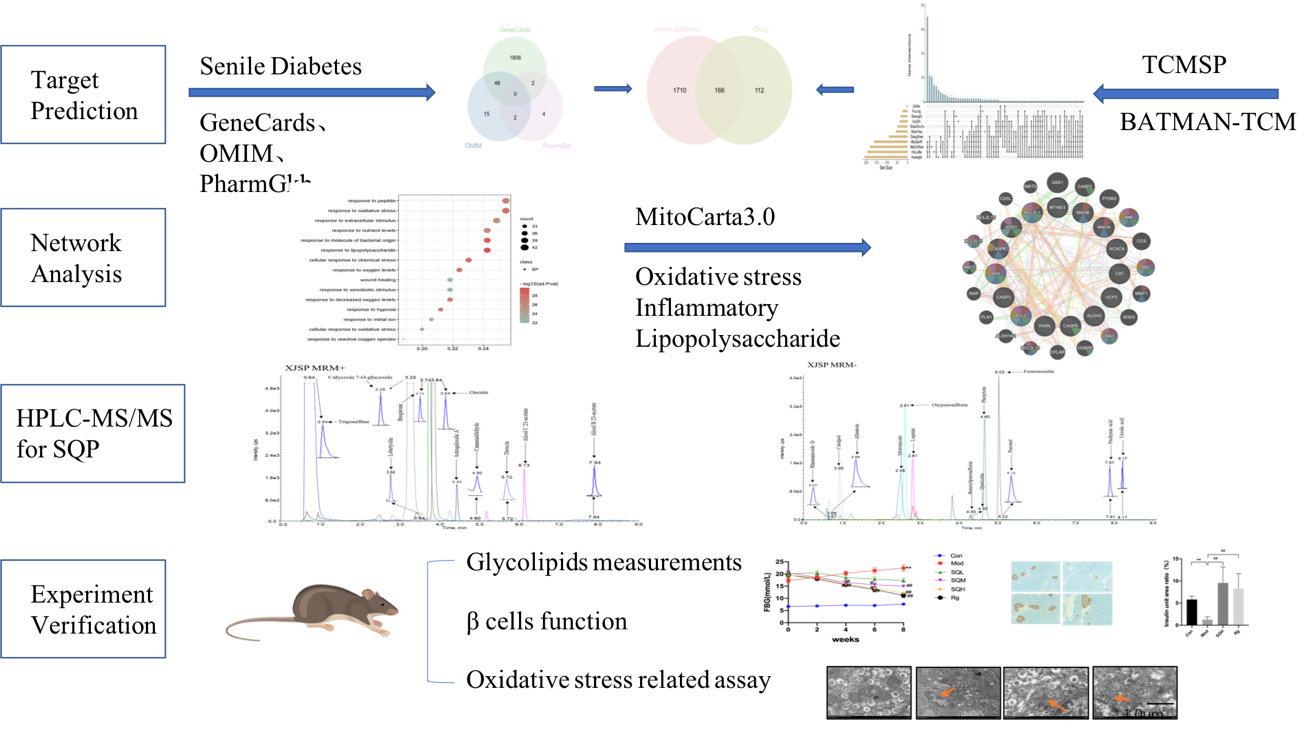
**

**Supplementary Figure 1.** Flowchart of ideas for this study.


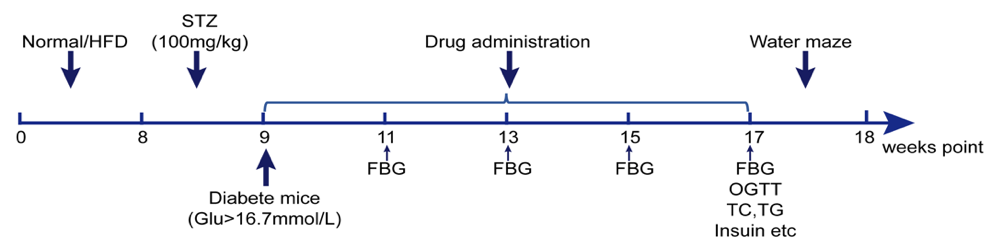


**STZ(30mg/kg)**

**Supplementary Figure 2.** SDM mice modeling and medication timeline.

**A**


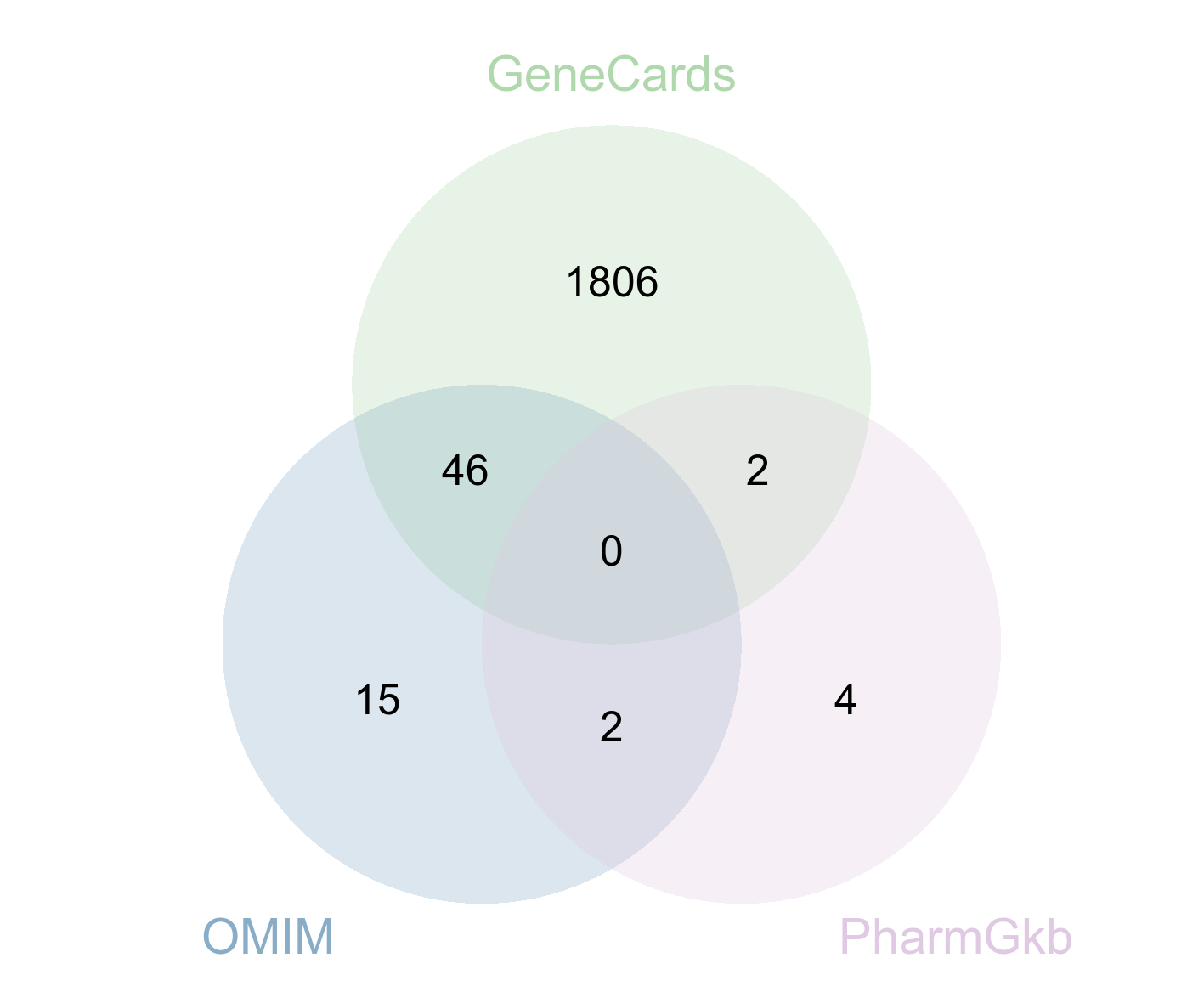


B


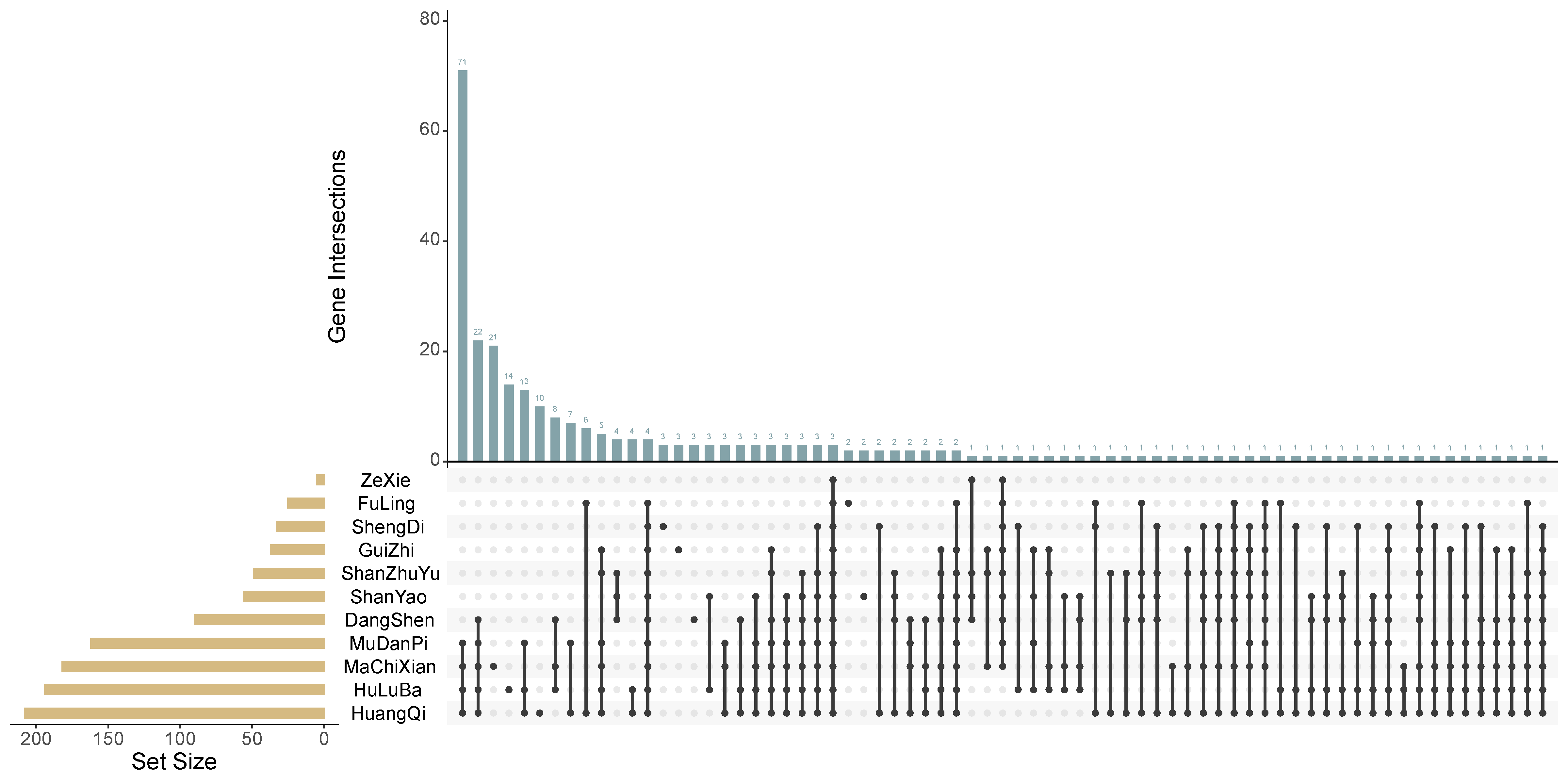


C


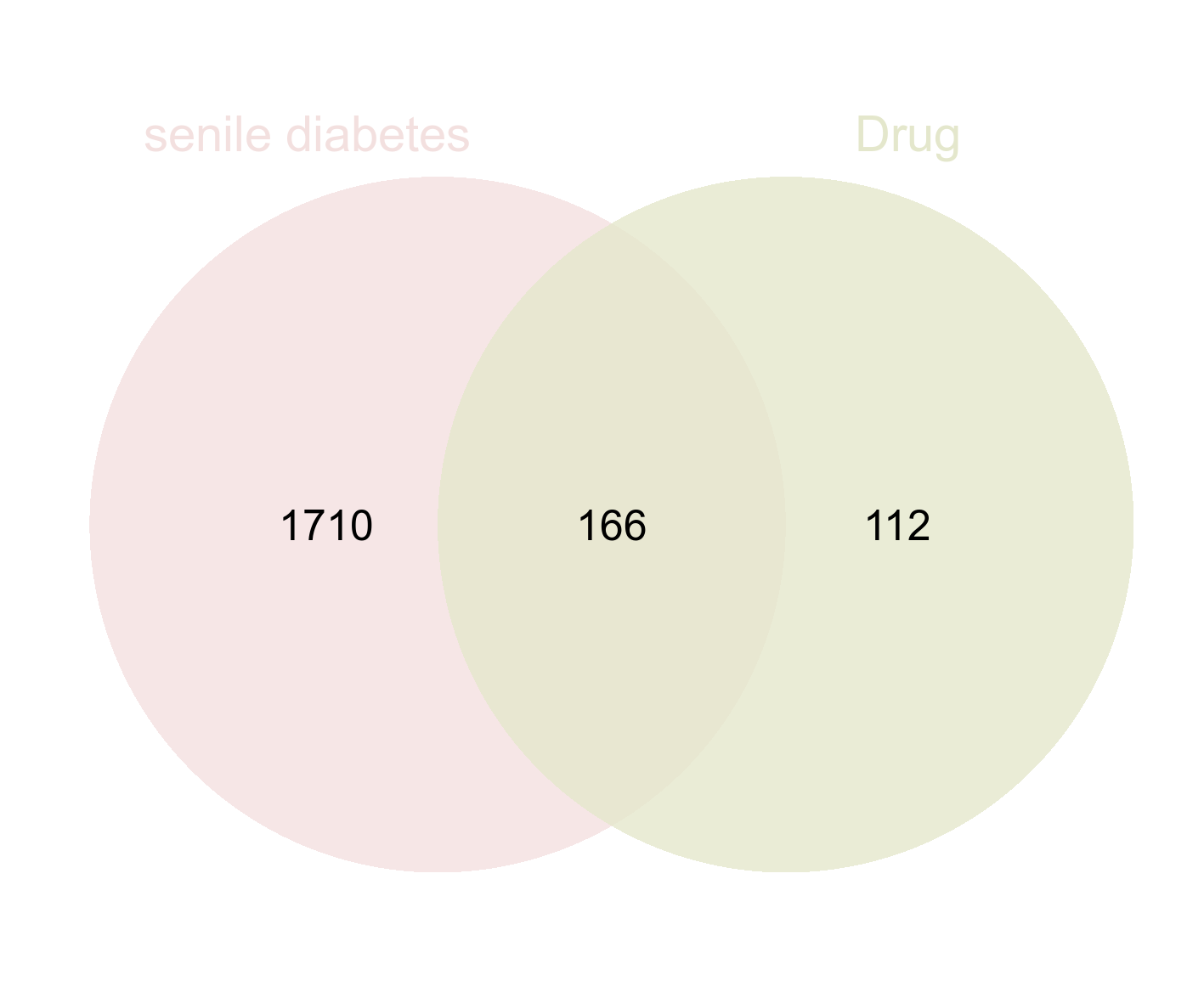


**Supplementary Figure 3.** (A) Three databases obtain the disease genes of diabetes in the elderly (B) Drug target genes of Shenqi Pills (C) Common target genes of SDM and SQP.


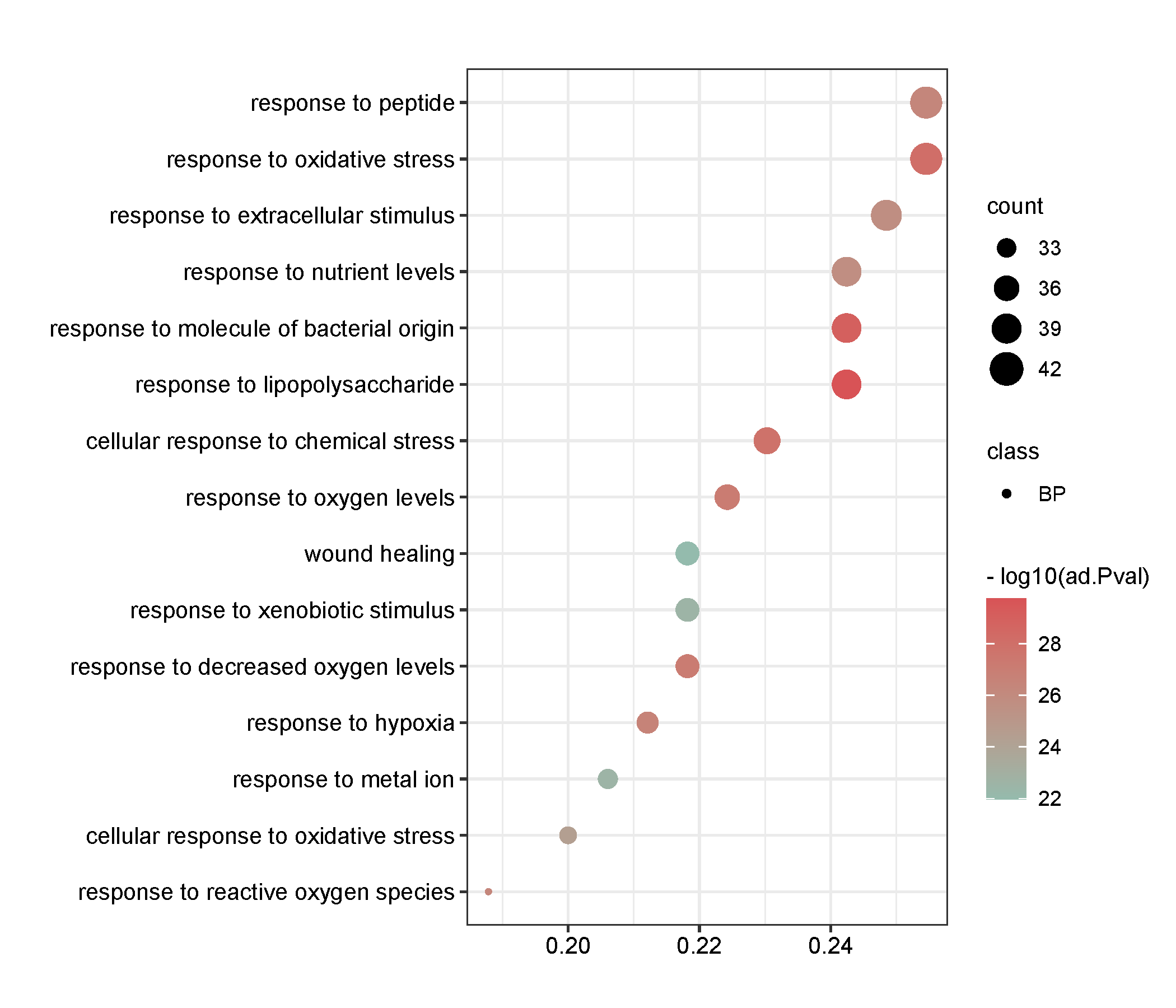


**Supplementary Figure 4.** GO enrichment analysis of BP-related common target genes of SDM and SQP.


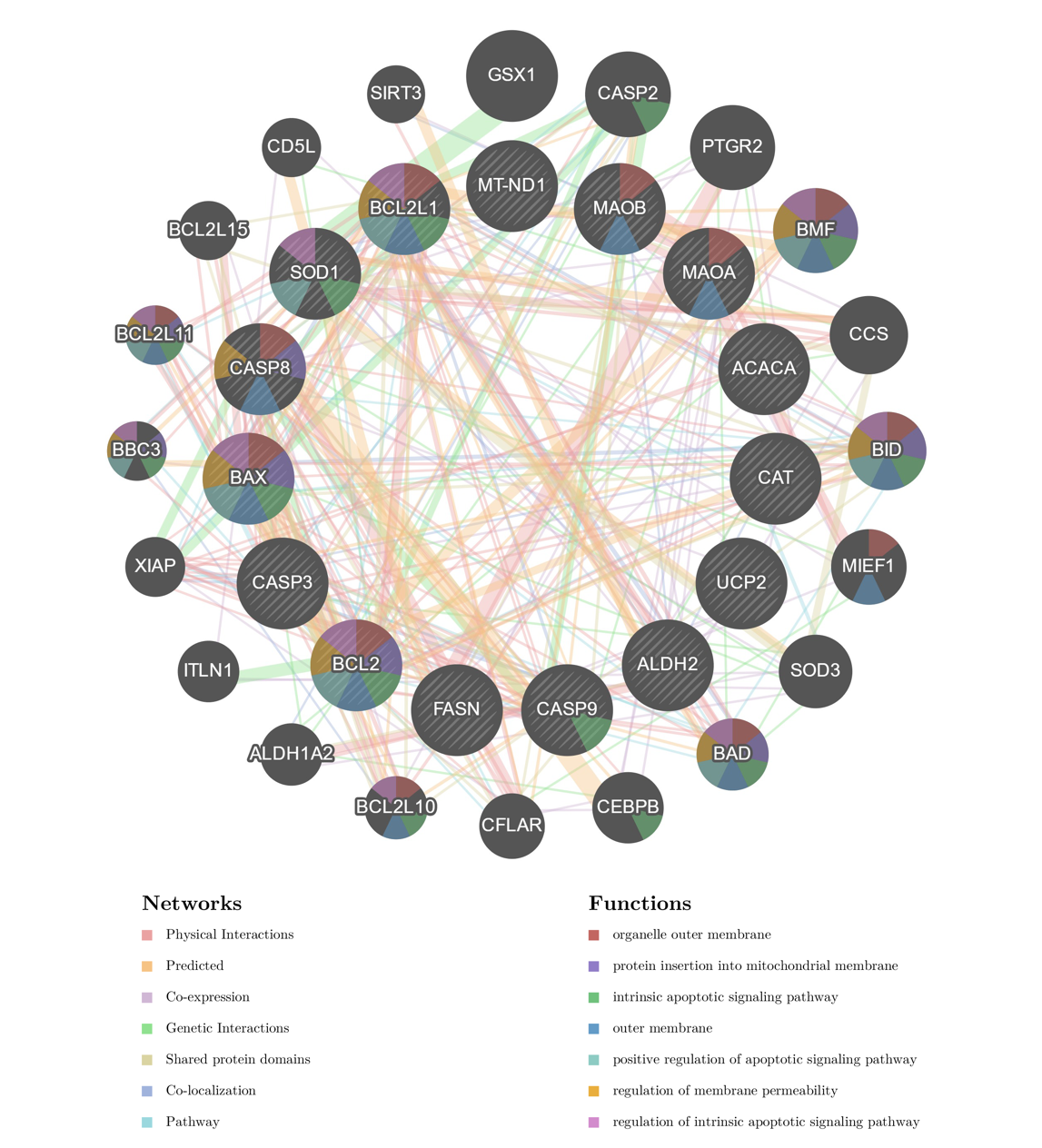


**Supplementary Figure 5.** Mitochondria-related drug therapeutic target functions.（Nodes in the network diagram represent potential target genes, and connections between nodes refer to interactions between proteins. The node's size and color will be scaled using the "degree" value.）

A


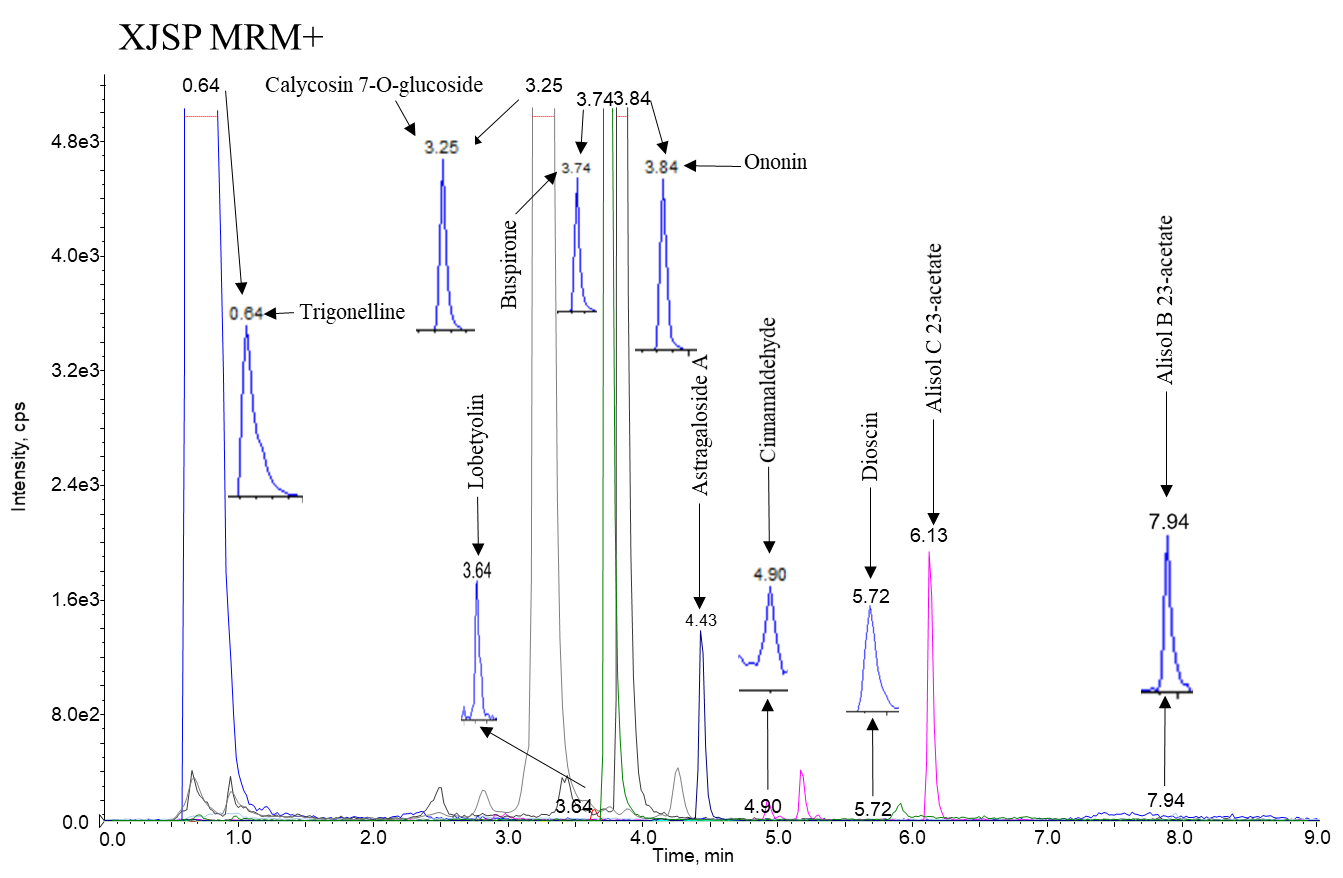


B


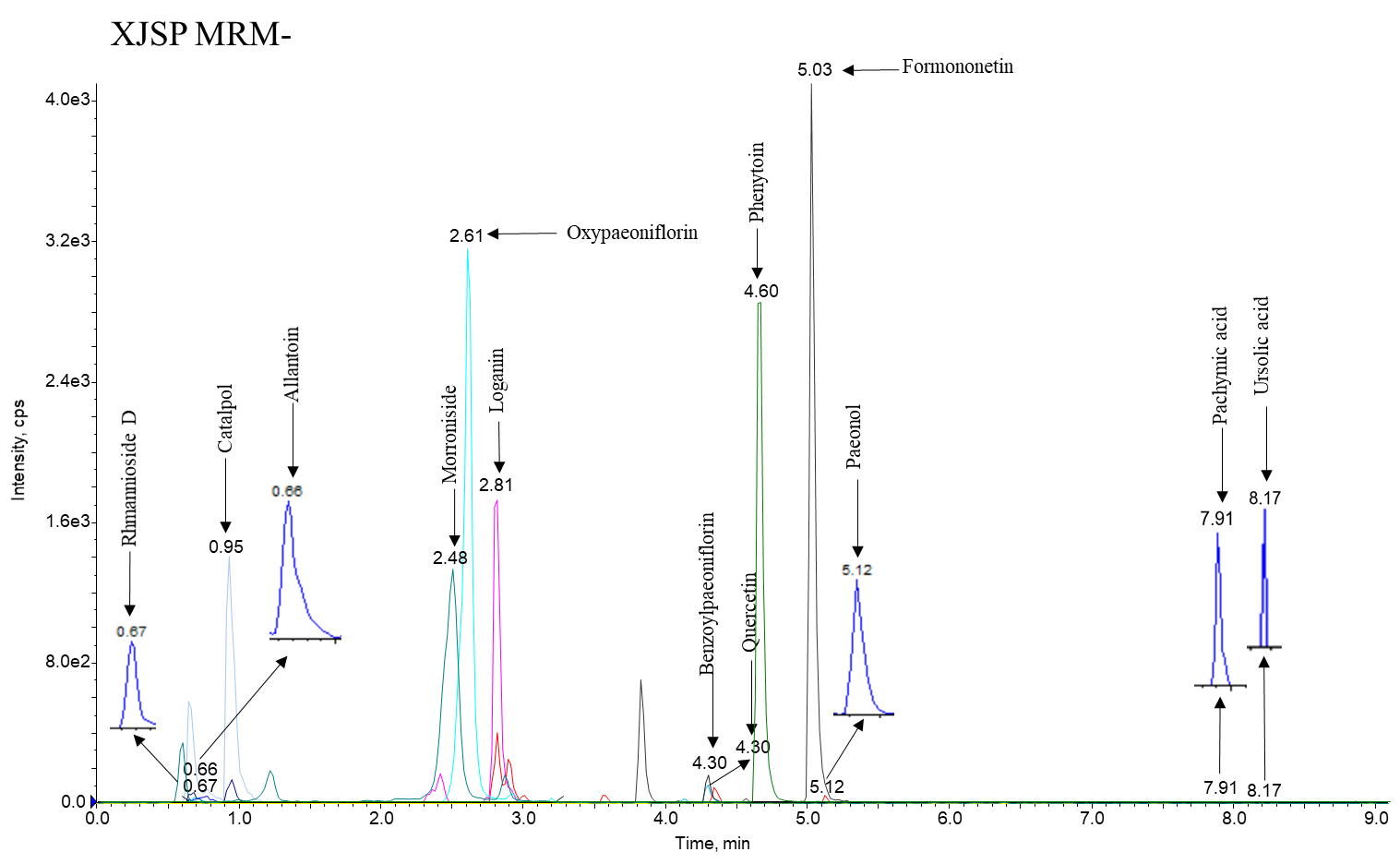


**Supplementary Figure 6.** Representative chromatograms of 21 compounds in SQP. (A) Positive ion mode chromatogram (B) Negative ion mode chromatogram.


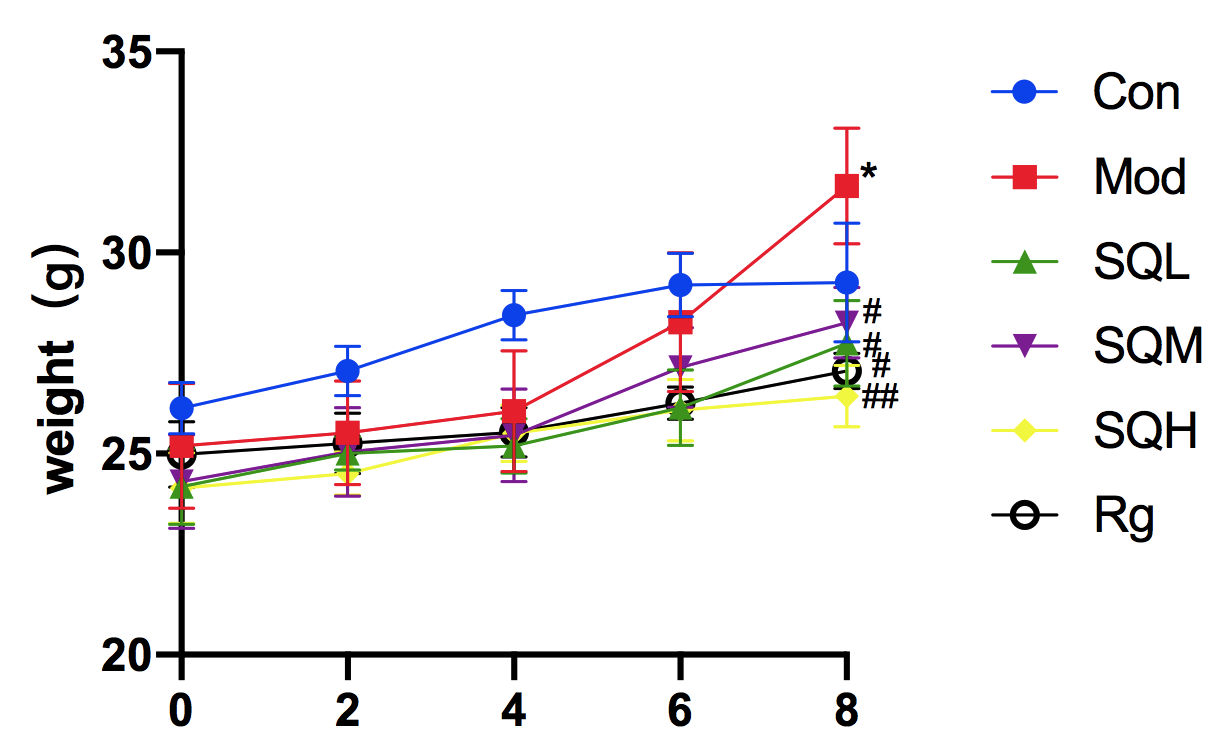


**Supplementary Figure 7.** Body weight of mice in each group at different time points. (Compared with Con，**P*<0.05，***P*<0.01；Compared with Mod，^#^*P*<0.05；^##^*P*<0.01)


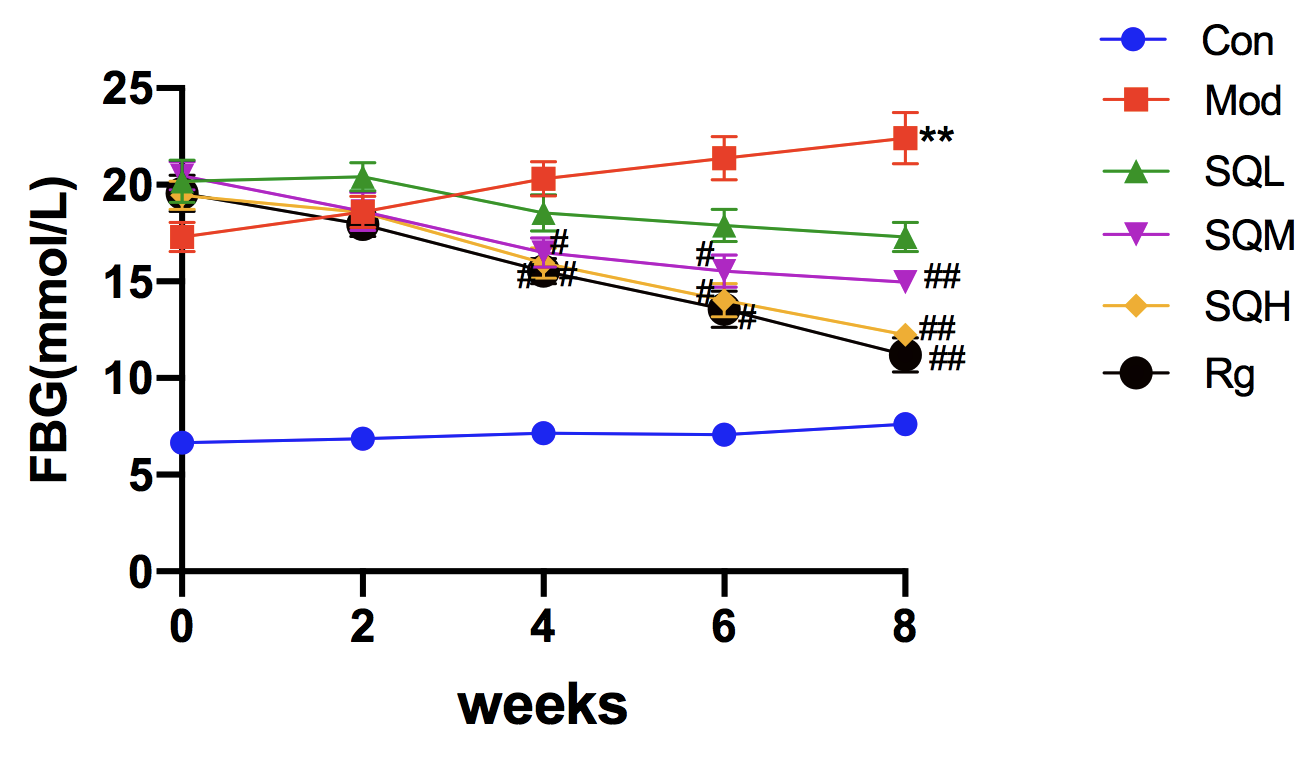


**Supplementary Figure 8.** FBG of mice in each group at different time points.

A B


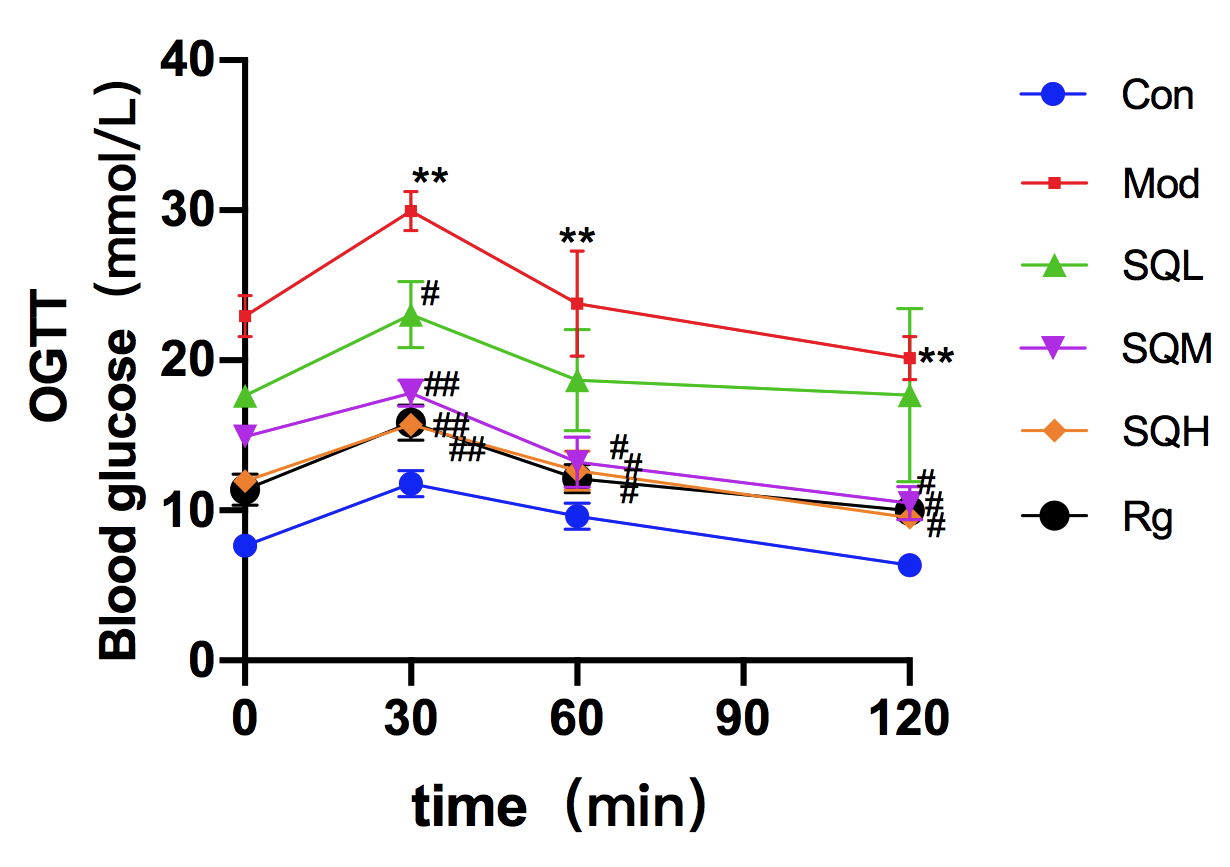

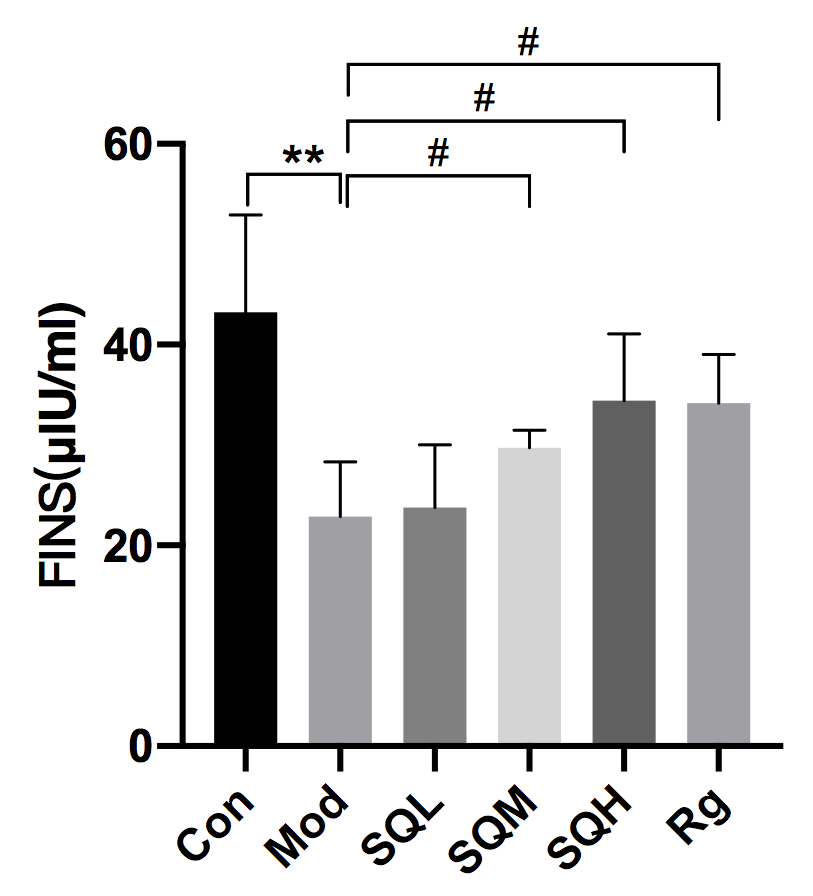


**Supplementary Figure 9.** Effect of SQP on OGTT and FINS in mice.（A）OGTT in mice（B）FINS in mice. (Compared with Con, ***P*<0.01；Compared with Mod，^#^*P*<0.05)

A B


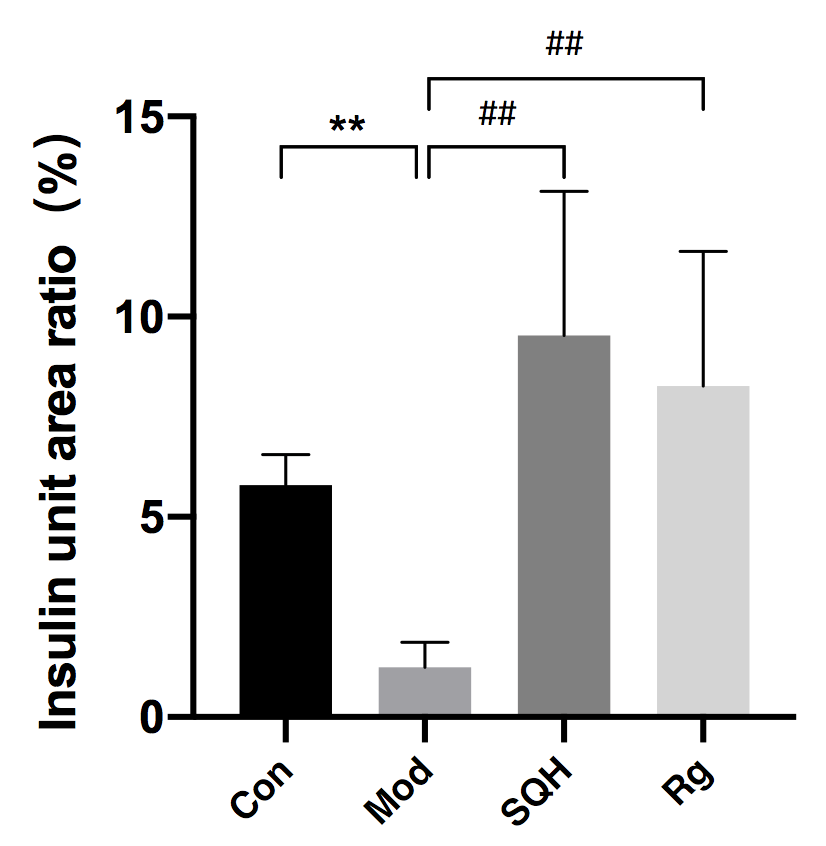

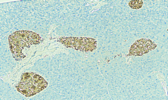

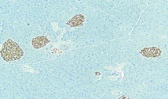

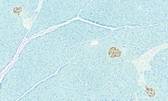


Con

Mod

SQH

Rg


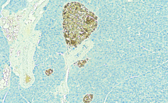


**Supplementary Figure 10.** Effects of insulin secretion levels in pancreatic tissue of mice（400×） (A) Histochemical staining results of insulin in pancreatic tissue of mice under high magnification (B) Statistics of insulin levels per unit area of pancreatic tissue in mice. (Compared with Con, ***P*<0.01；Compared with Mod，^##^*P*<0.01)

A


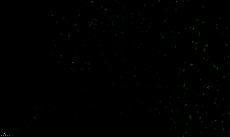

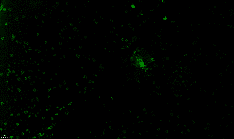

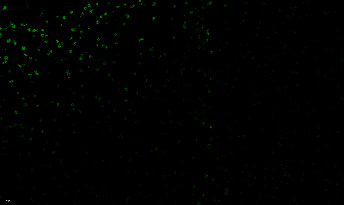

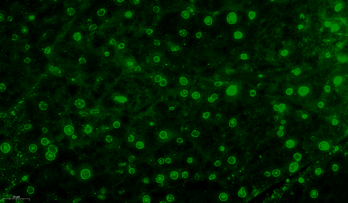


FITC

DAPI

Merge


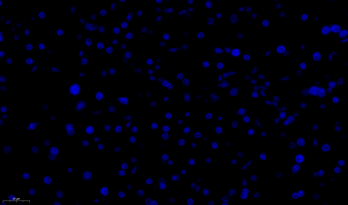

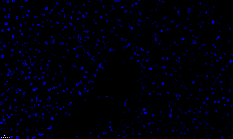

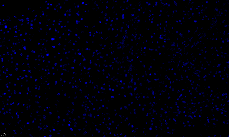

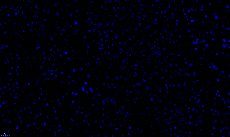

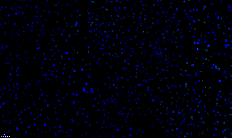

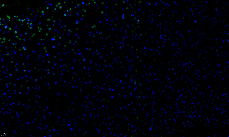

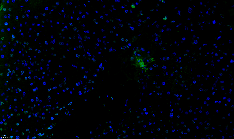

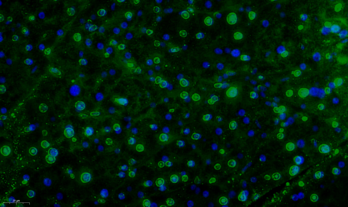


Con

Mod

SQH

Rg


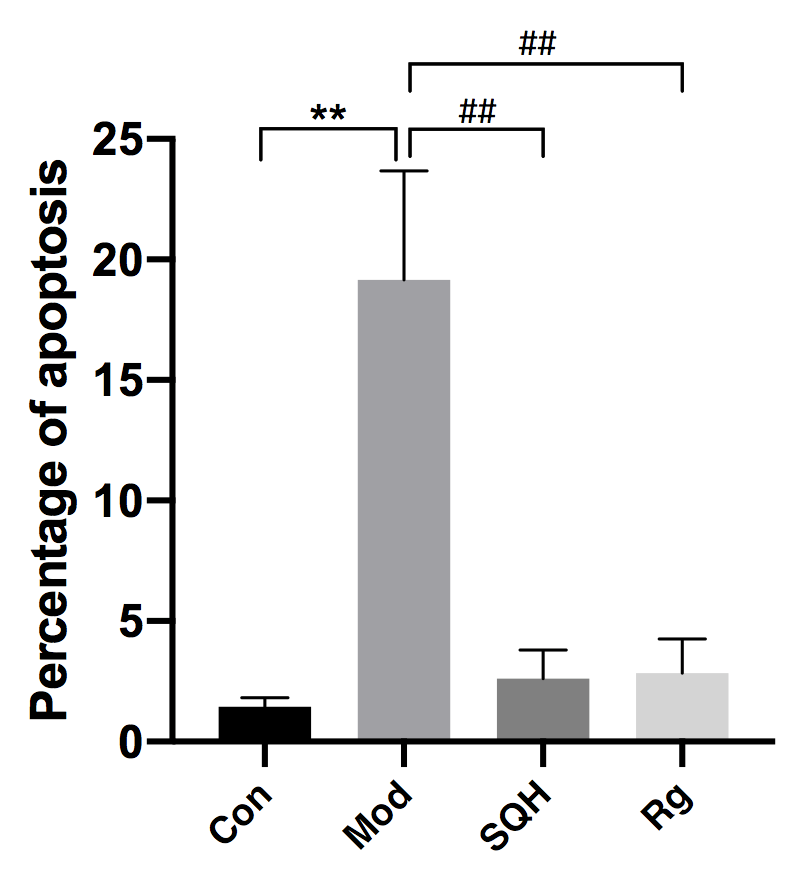
B

**Supplementary Figure 11.** Apoptosis rate of pancreatic β cells in mice（400×）(A) Staining results of pancreatic β-cells in mice under a fluorescence microscope (B) Statistics of apoptosis of pancreatic β-cells in mice. (Compared with Con, ***P*<0.01；Compared with Mod，^##^*P*<0.01)

A B C


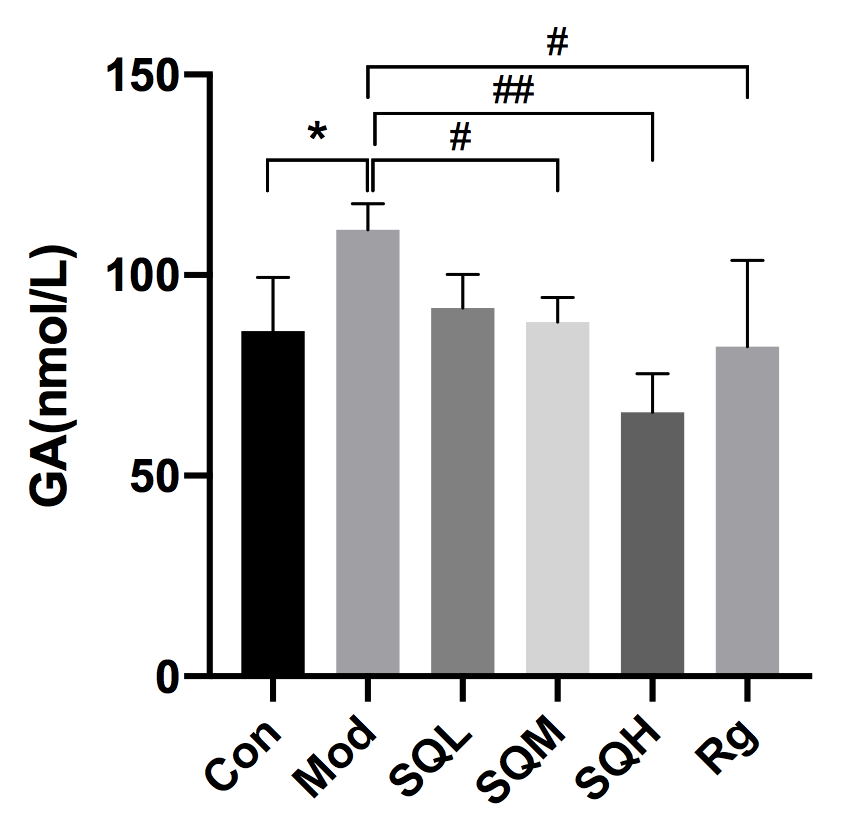

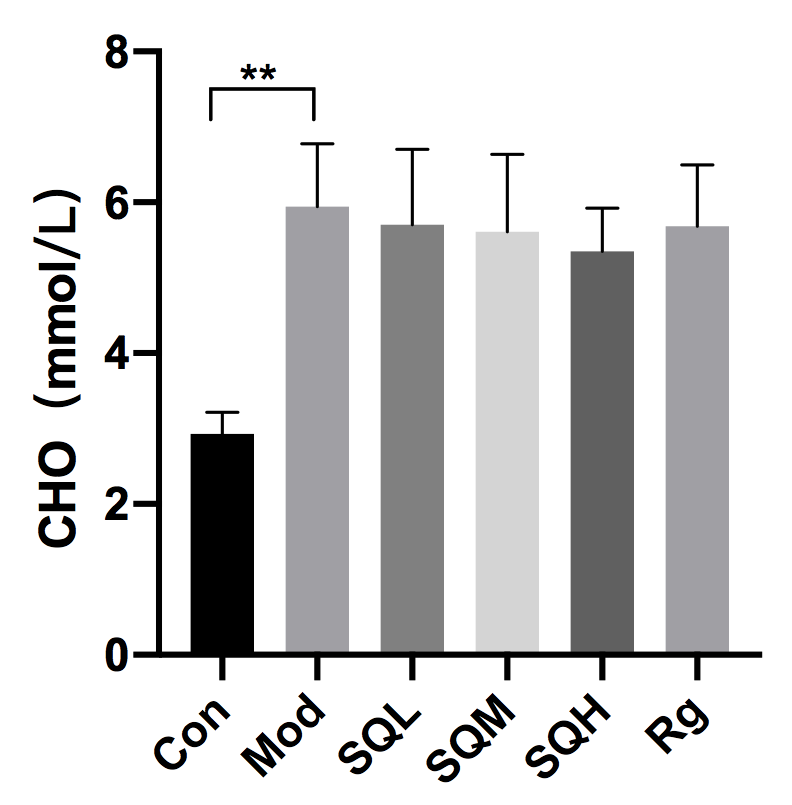

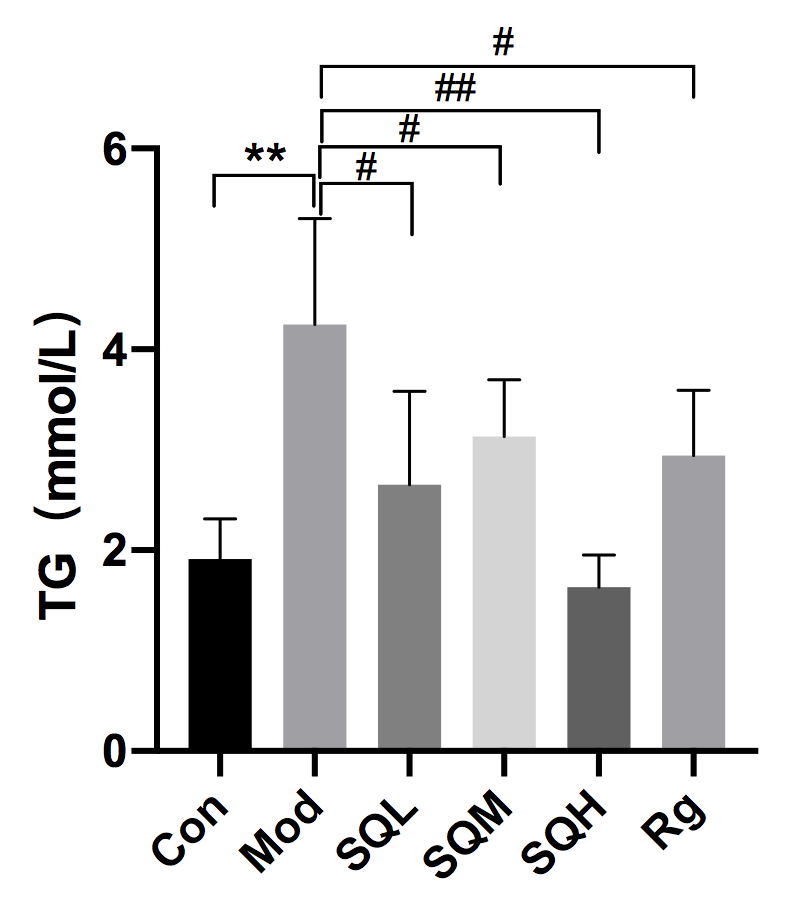


D E F


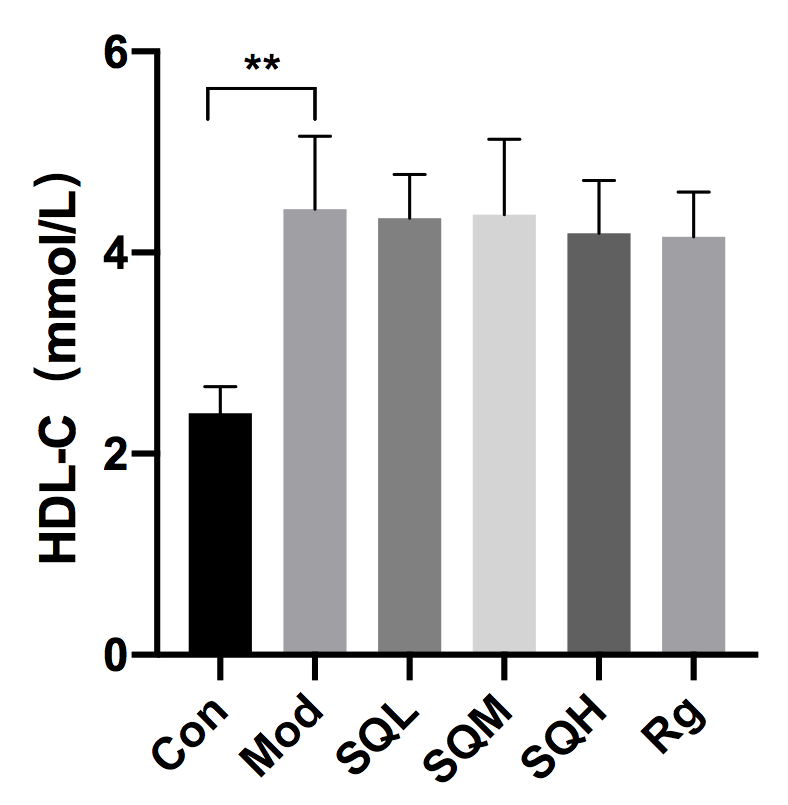

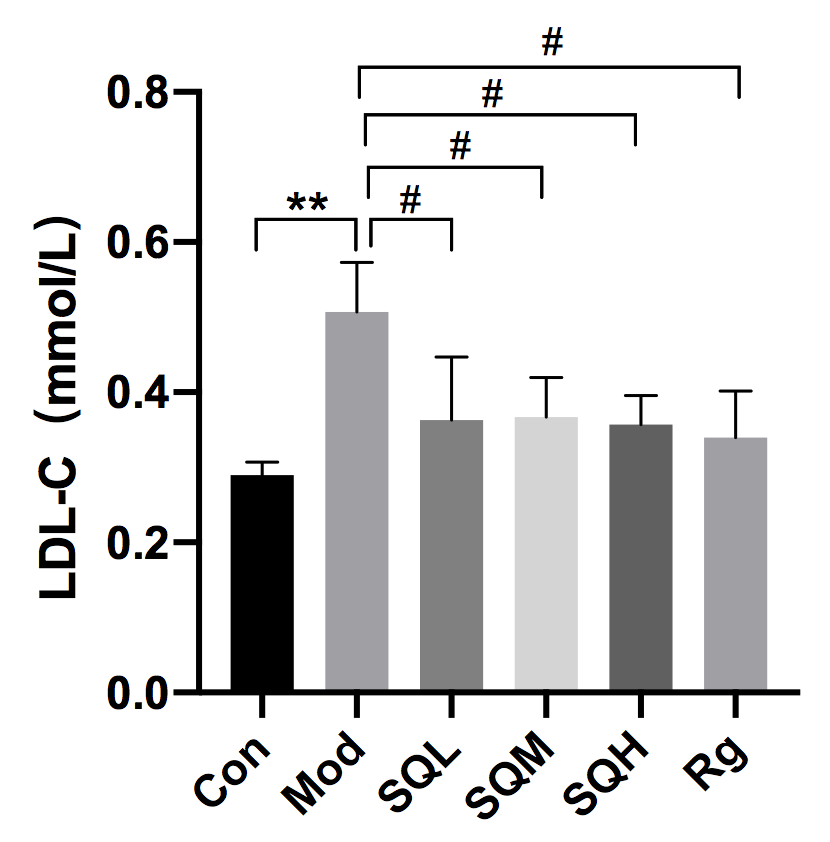

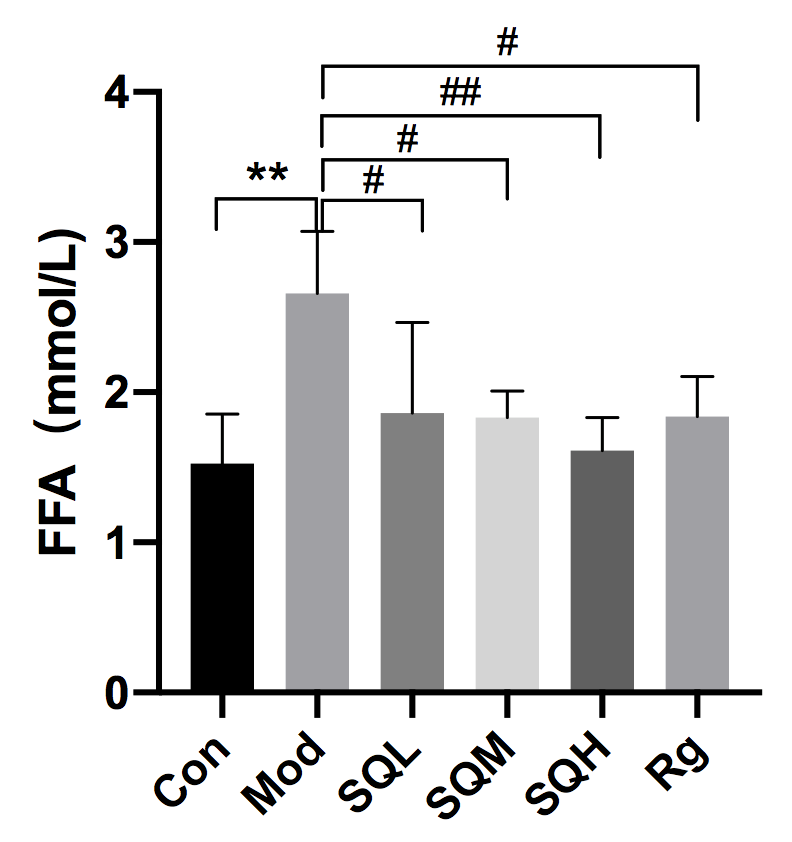


**Supplementary Figure 12.** GA, CHO, TG, HDL-C, LDL-C and FFA levels of mice in each group (A) GA in mice; (B-E) Four blood lipids in mice; (F) FFA in mice. (Compared with Con，**P*<0.05，***P*<0.01；Compared with Mod，^#^*P*<0.05；^##^*P*<0.01)

A B


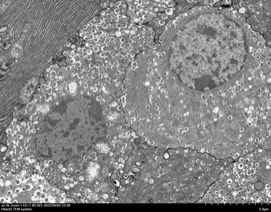

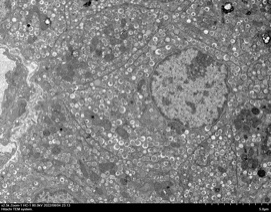

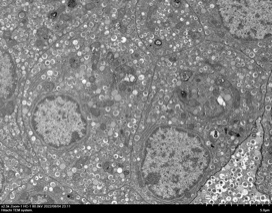

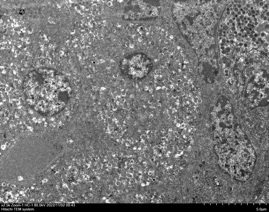


Con

Mod

SQH

Rg

5.0um


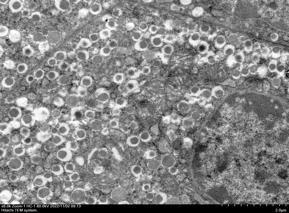

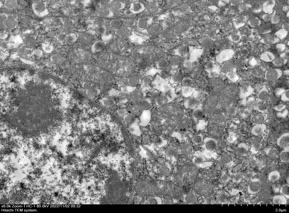

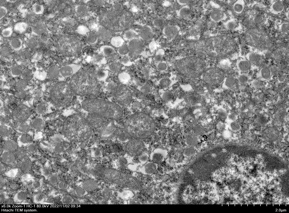

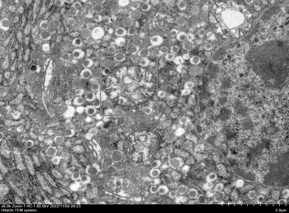


Con

Mod

Rg

SQH

2.0um

C


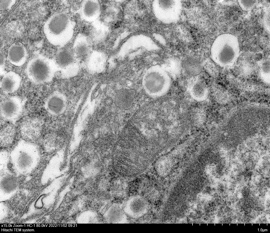

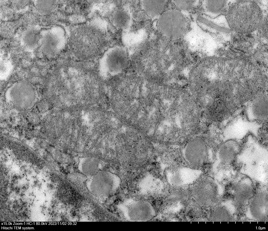

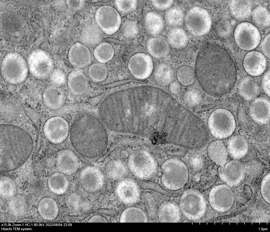

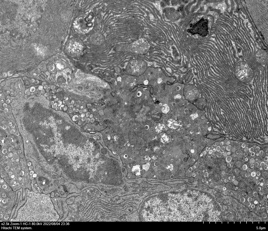


Con

Mod

SQH

Rg

1.0um

**Supplementary Figure 13.** Transmission electron microscopy observation of the ultrastructure of pancreatic β cells. (A) Nucleus structure of pancreatic islet β cells (B) Cytoplasmic granule structure of pancreatic islet β cells (C) Mitochondrial structure of pancreatic islet β cells （The arrows in Figure A point to the pyknotic nuclei of islet β cells. The boxes in Figure B indicate reduced cytoplasmic granules. The arrows in the Mod in Figure C point to mitochondrial cristae with broken morphology, and the arrows in the SQH and Rg point to the presence of normal mitochondrial cristae morphology.）

A


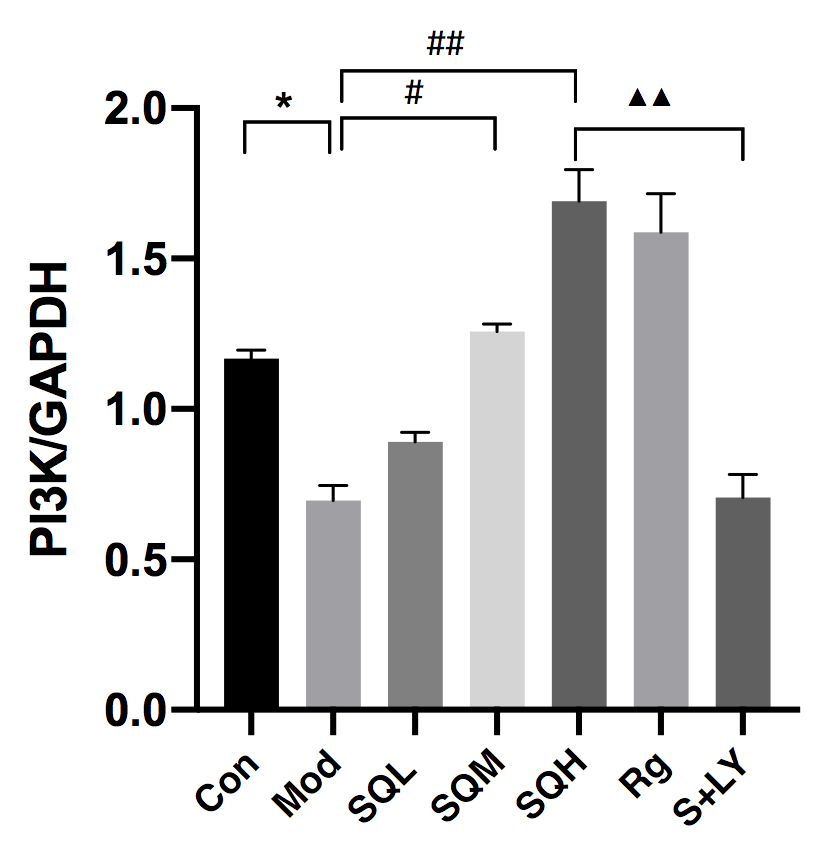

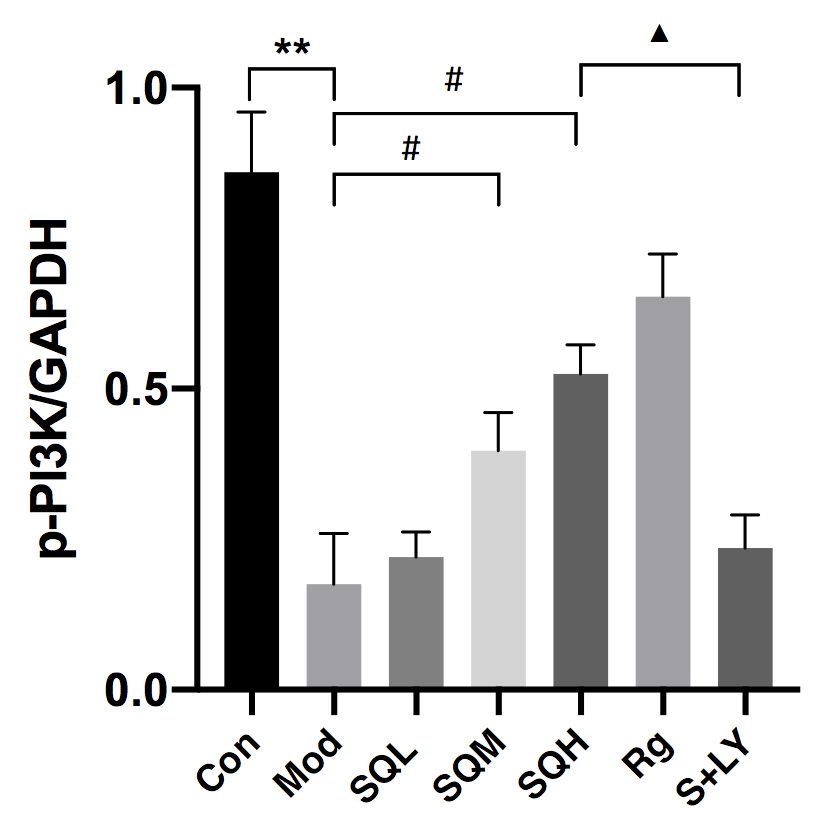


p-PI3K

85KD


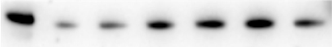


Con

Mod

SQL

Rg

SQM

SQH


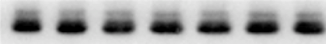

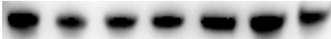


GAPDH

PI3K

85KD

37KD

S+LY

B

Con

Mod

SQL

Rg

SQM

SQH


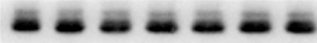


GAPDH

Akt

60KD

37KD


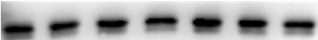

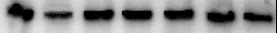


60KD

p-Akt


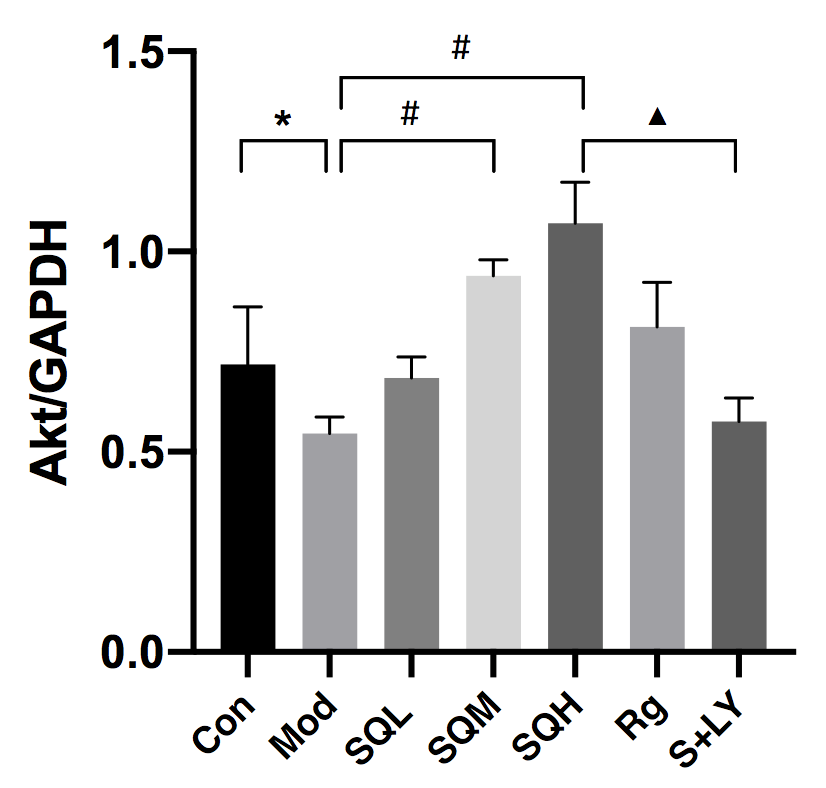

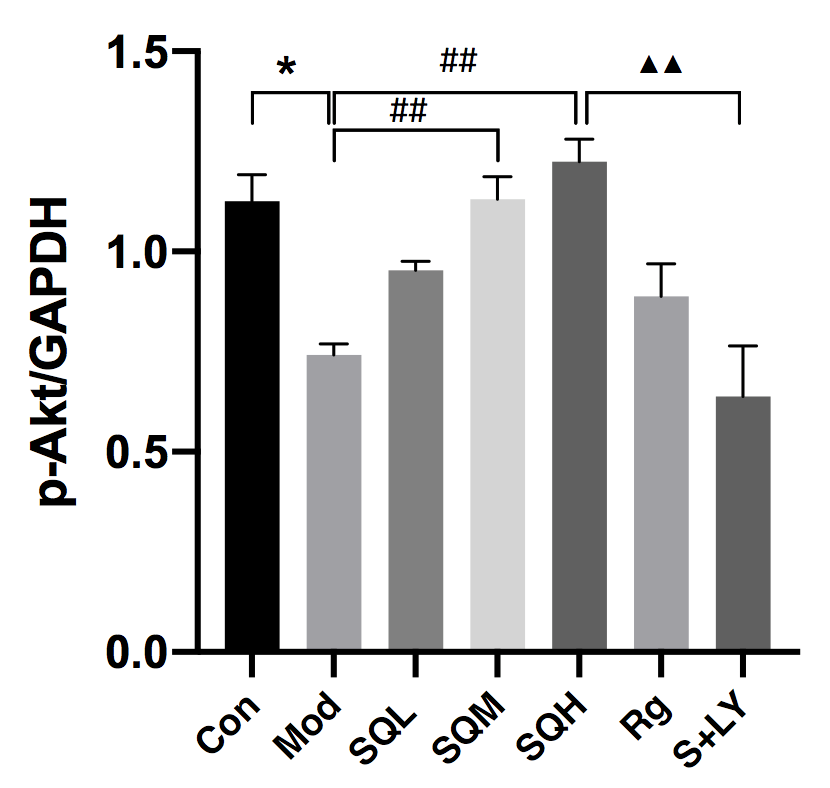


S+LY

C

S+LY

S+LY

Con

Mod

SQL

Rg

SQM

SQH


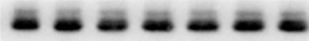


GAPDH

GSK-3β

47KD

37KD


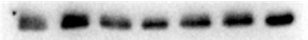

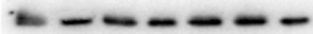


p-GSK-3β

47KD


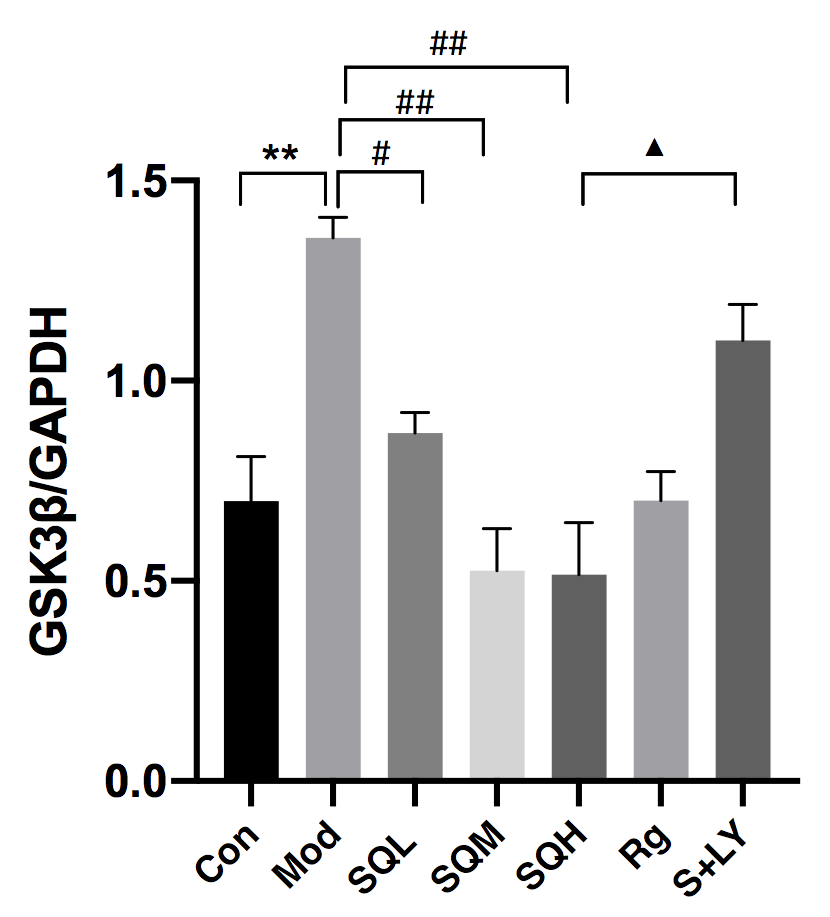

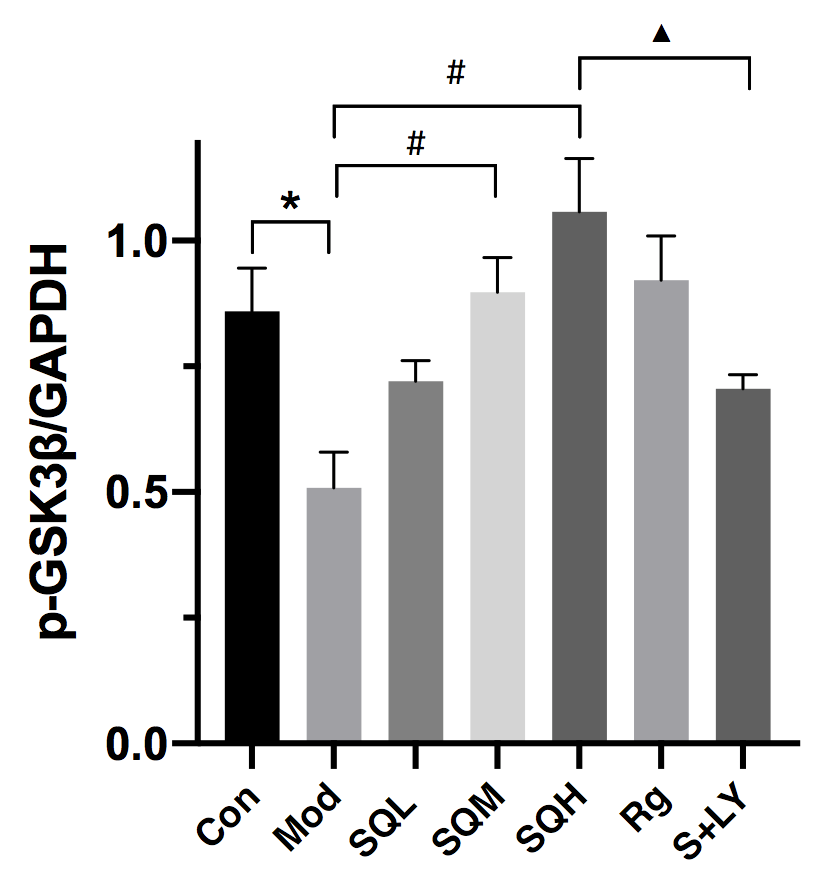


**Supplementary Figure 14.** The expression of PI3K-Akt-GSK-3β signaling axis protein.（A）The expression of PI3K/p-PI3K（B）The expression of Akt/p-Akt（C）The expression of GSK-3β/p-GSK-3β ((Compared with Con, **P*<0.05, ***P*<0.01；Compared with Mod，^#^*P*<0.05, ^##^*P*<0.01; Compared with SQH，^▲^*P*<0.05，^▲▲^*P*<0.01.)

## Supplementary Tables

| botanical drug | Unit (g) | botanical drug | Unit (g) |
| --- | --- | --- | --- |
| Rehmannia glutinosa | 24 | Moutan cortex | 9 |
| Rhizoma dioscoreae | 12 | Cinnamomum cassia | 3 |
| Cornus officinalis | 12 | Codonopsis pilosula | 9 |
| Wolfiporia extensa | 9 | Astragalus membranaceus | 12 |
| Alisma orientalis | 9 | Common fenugreek seed | 6 |
| Purslane | 9 |  |  |

**Supplementary Table 1.** SQP Dosage (dosage per dose of medication).

| compounds | concentrations（μg/g） | | | |
| --- | --- | --- | --- | --- |
|  | SQP 1-1 | SQP 1-2 | SQP 2-1 | SQP 2-2 |
| Rhmannioside D | 490 | 458 | 361 | 449 |
| Catalpol | 5100 | 4730 | 4200 | 3990 |
| loganin | 308 | 292 | 323 | 264 |
| Monogside | 291 | 289 | 293 | 283 |
| Ursolic acid | 1.83 | 2.30 | 0.656 | 0.930 |
| Cinnamaldehyde | 615 | 741 | 864 | 964 |
| Trigonelline | 266 | 280 | 489 | 508 |
| Lobetyolin | 32.1 | 31.5 | 37.7 | 35.9 |
| Alisol B 23-acetate | 35.0 | 31.6 | 52.2 | 64.3 |
| Alisol C 23-acetate | 5.83 | 7.04 | 10.9 | 13.9 |
| Allantoin | 201 | 196 | 413 | 313 |
| Dioscin | 24.8 | 17.2 | 17.7 | 16.0 |
| Calycosin 7-*O*-glucoside | 52.3 | 55.4 | 61.0 | 64.5 |
| Formononetin | 10.6 | 9.95 | 18.9 | 18.4 |
| Ononin | 50.3 | 59.1 | 107 | 122 |
| Benzoylpaeoniflorin | 330 | 320 | 267 | 253 |
| Oxypaeoniflorin | 254 | 240 | 189 | 189 |
| Paeonol | 308 | 366 | 328 | 252 |
| Astragaloside A | 117 | 141 | 133 | 158 |
| Quercetin | 3.29 | 2.36 | 3.12 | 2.19 |
| Poria acid | 17.6 | 16.8 | 4.48 | 6.47 |

**Supplementary Table 2.** Actual measured concentrations of 21 compounds in SQP.
